# COVID-19 case prediction using emotion trends *via* Twitter emoji analysis: A case study in Japan

**DOI:** 10.3389/fpubh.2023.1079315

**Published:** 2023-03-14

**Authors:** Vu Tran, Tomoko Matsui

**Affiliations:** ^1^Risk Analysis Research Center, The Institute of Statistical Mathematics, Tokyo, Japan; ^2^Department of Statistical Modeling, The Institute of Statistical Mathematics, Tokyo, Japan

**Keywords:** COVID-19, emotion, emoji, anomaly detection, social media, Twitter

## Abstract

**Introduction:**

The worldwide COVID-19 pandemic, which began in December 2019 and has lasted for almost 3 years now, has undergone many changes and has changed public perceptions and attitudes. Various systems for predicting the progression of the pandemic have been developed to help assess the risk of COVID-19 spreading. In a case study in Japan, we attempt to determine whether the trend of emotions toward COVID-19 expressed on social media, specifically Twitter, can be used to enhance COVID-19 case prediction system performance.

**Methods:**

We use emoji as a proxy to shallowly capture the trend in emotion expression on Twitter. Two aspects of emoji are studied: the surface trend in emoji usage by using the tweet count and the structural interaction of emoji by using an anomalous score.

**Results:**

Our experimental results show that utilizing emoji improved system performance in the majority of evaluations.

## 1. Introduction

Almost 3 years have passed since the beginning of the worldwide COVID-19 pandemic at the end of 2019. The pandemic has been causing severe global problems in many aspects of life. During a pandemic, information availability is critical to helping people get through the hardships. Social media in particular has been a prevalent source of COVID-19 related information (1, 2). A questionnaire survey of 1,003 US-based adults by Neely et al. (1) showed that 76% of the respondents relied at least somewhat on social media for COVID-19 related information, that 59% read COVID-19 related information on social media at least once per week, and that 63.6% were unlikely to check facts with a healthcare professional. A cross-sectional study among university students in Germany by Dadaczynski et al. (2) showed that 37.6% (5,302/14,092) of the respondents used social media occasionally or frequently to search for information on COVID-19 and related issues.

Social media has been shown to reflect social mental states. An analysis of Facebook posts by Settanni and Marengo (3) revealed that, overall, the expression of negative emotions positively correlated with anxiety, depression, and stress symptoms and that negative emoji usage positively correlated with anxiety symptoms. Park et al. (4) found that the use of words related to negative emotions and anger significantly increased among Twitter users with major depressive symptoms compared with those otherwise. Wald et al. (5) showed that the traits in the Big 5 Personality Index (6) (agreeableness, conscientiousness, extroversion, neuroticism, and openness) and those in the Dark Triad (7) (psychopathy, Machiavellianism, narcissism) could be predicted for social media users from their Twitter posts (“tweets”) with rather good accuracy (area under the ROC curve of 0.736).

Furthermore, several studies have revealed that social media users tend to exhibit negative emotions toward COVID-19 progression. Wheaton et al. (8) showed that “time interacting with social media did predict symptoms of depression and stress, but not anxiety or OCD (obsessive-compulsive disorder) symptoms.” Arora et al. (9) showed that “people with a negative sentiment are more susceptible to addictive use of social media.” An analysis of Twitter data for February, May, and June, 2020, by Kaur et al. (10) showed that the highest percentage of tweets belonged in the “negative” category. Toriumi et al. (11) showed in their analysis using Twitter data for Japan that social emotions toward COVID-19 from February to April, 2020, were mainly influenced by “fear.” Another analysis of Twitter data by Dyer and Kolic (12) revealed “evidence of psychophysical numbing: Twitter users increasingly fixate on mortality, but in a decreasingly emotional and increasingly analytic tone.” Social media can thus cause severe mental health problems including high levels of stress, anxiety, and contagious fear (13, 14). Furthermore, high levels of COVID-19 misinformation and fake news can exaggerate perceived risk (15). Nevertheless, regulating fake news content is still a challenging problem (16).

This study was aimed at determining whether COVID-19 related emotion trends on Twitter can enhance COVID-19 case prediction in Japan. The core idea is that emoji usage is a potential proxy that captures user emotions from user contents. Emoji are digital images depicting simple but eye-catching illustrations including facial expressions (e.g., 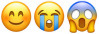) for expressing emotional messages effectively as social media users share a common understanding of emoji and use them on social media as non-verbal communication cues to assist communication (17–20). Several studies have focused on capturing emotion from texts including posts on Twitter (“tweets”), for example, sentiment analysis (21), and emotion analysis (22). However, accurately understanding emotional tweets by using full-text analysis is still a challenging task. Therefore, shallow emotion analysis using emoji is instead an attractive approach, especially since it is applicable in multilingual contexts. Our utilization of emoji was done from two perspectives: the surface trend of emoji by tweet count analysis and the structural interaction of emoji by anomalous score computation. Furthermore, we built an ensemble of long short-term memory (LSTM) models (23) to perform the target COVID-19 case prediction task, which is described in the next section.

Our data collection shows that the top popular emoji used in Japanese tweets are facial expressions that representing several emotions including happiness, sadness, fear, and anger. From the results by the previous studies, we hypothesize that the social media emotion reaction relates to emotional behavioral changes influencing COVID-19 epidemic progression, so the social media emotion reaction captured from the data of those top popular emoji can help improve our COVID-19 case prediction system.

Even though there are studies of COVID-19 case prediction systems that use data from social media, particularly Twitter, the potential of emoji analysis in enhancing COVID-19 case prediction system performance has not yet been well explored. Yousefinaghani et al. (24) collected tweets related to COVID-19 symptoms for building a system to predict COVID-19 outbreaks. Azzaoui et al. (25) performed tweet analysis using common text analysis techniques like term frequency-inverse document frequency without explicit consideration of emotion analysis. Chew et al. (26) mentioned the use of Twitter data as a source of emotional responses toward COVID-19, but they did not perform emotion analysis on the data. Tran and Matsui (27) considered the tweet count of emoji-using tweets but did not perform a breakdown analysis of each emoji.

## 2. Materials and methods

**Figure 1:**
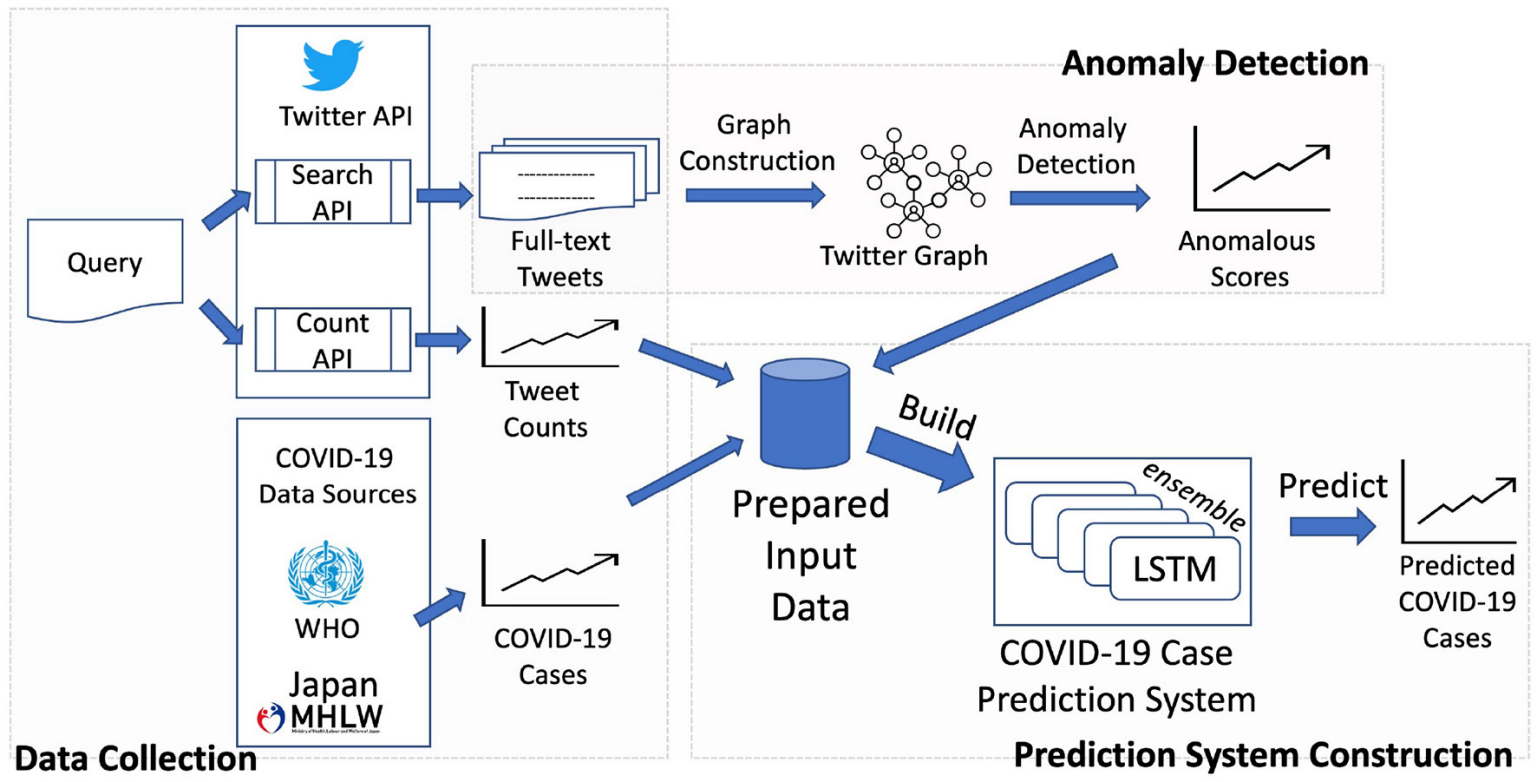
General illustration of our framework.

In this section, we describe in details of our approach to building and evaluating our framework for predicting COVID-19 cases given the past cases and COVID-19 related Twitter data. Our framework illustrated in Figure 1 consists of three major processes: Data Collection (Section 2.1), Anomaly Detection (Section 2.3), and Prediction System Construction (Section 2.4). In the “Data Collection” process, we collect COVID-19 related tweets containing emoji to obtain the social media emotion trends *via* tweet count and detect anomaly in those trends by analyzing full-text tweets in the “Anomaly Detection” process. After preparing our necessary input data including the social media data and COVID-19 case data from official data sources, we build our COVID-19 case prediction system utilizing LSTM, a deep neural network for time-series modeling, and “ensemble of the best,” as described in the “Prediction System Construction” process. The framework is developed with and evaluated on almost 3 years' worth of data from January 2020 to October 2022.

### 2.1. Data collection

We considered Twitter as the social media platform for this study because of these two main points: 1) Twitter is a top popular social media platform in Japan and 2) Twitter promotes public social media engagements. According to Statista^1^, Japan ranks 2nd in the number of Twitter users in 2022 after US. According to BigBeat^2^, Twitter with more than 50 million users ranks 2nd after LINE which focuses on private engagements while Twitter promotes public engagements which can be easily participated by strangers. On top of that, Twitter provides API for researchers to access full historical data.

For the case study in Japan, the data used consisted of COVID-19 infection data and COVID-19 related tweets in Japanese. The COVID-19 infection data were publicly provided by the Japanese Ministry of Health, Labor and Welfare.^3^ The tweet data were collected using the Twitter API (version 2) with academic research access by matching a set of predefined keywords and emoji whose majority are facial expressions showing several kind of emotions: happiness, sadness, fear, anger, etc. (see Supplementary material) with the top 3 emoji are crying, sweating (may also be seen as raindrop) and smiley emoji. We chose the keywords based on observing Twitter trending phrases related to COVID-19 in Japan with four categories: general posts about COVID-19, posts about the reporting of the number of infections, posts about vaccination, and posts about Japanese government policy—the emergency state declaration and the spread-prevention policy. The details of the settings are shown in Table 1. Since location-tag is off by default and mostly not turned on by users, to select tweets in Japanese, we used the “lang” parameter provided by Twitter API with the language code “ja.” In the period from 2020/01/01 to 2022/09/30, we collected more than 20 million tweets in total. Beside the COVID-19 related tweets, we performed a count of all tweets that matched a predefined set of emoji. The total count was 8 billion for the same period.

**Table 1:** Tweet data collection setting.

**Query**	**Average daily no. of Tweets**
must contain at least one of {新型コロナ, コロナ感染, コロナ禍, コロナワクチン, 緊急事態宣言, まん延防止, 感染者}, and must contain emoji (e.g., 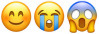 ), in the top 30 emoji (list of the emoji shown in Supplementary material) (*translation: [new-variant corona, corona infection, corona disaster, corona vaccine, emergency declaration, spread prevention, infected person/people]*)	29,484

The keywords were selected based on our observation of the trending keywords on Twitter which associate with reactions over COVID-19 news, COVID-19 case reporting, COVID-19 vaccination and government strong restriction policies.

**Figure 2:**
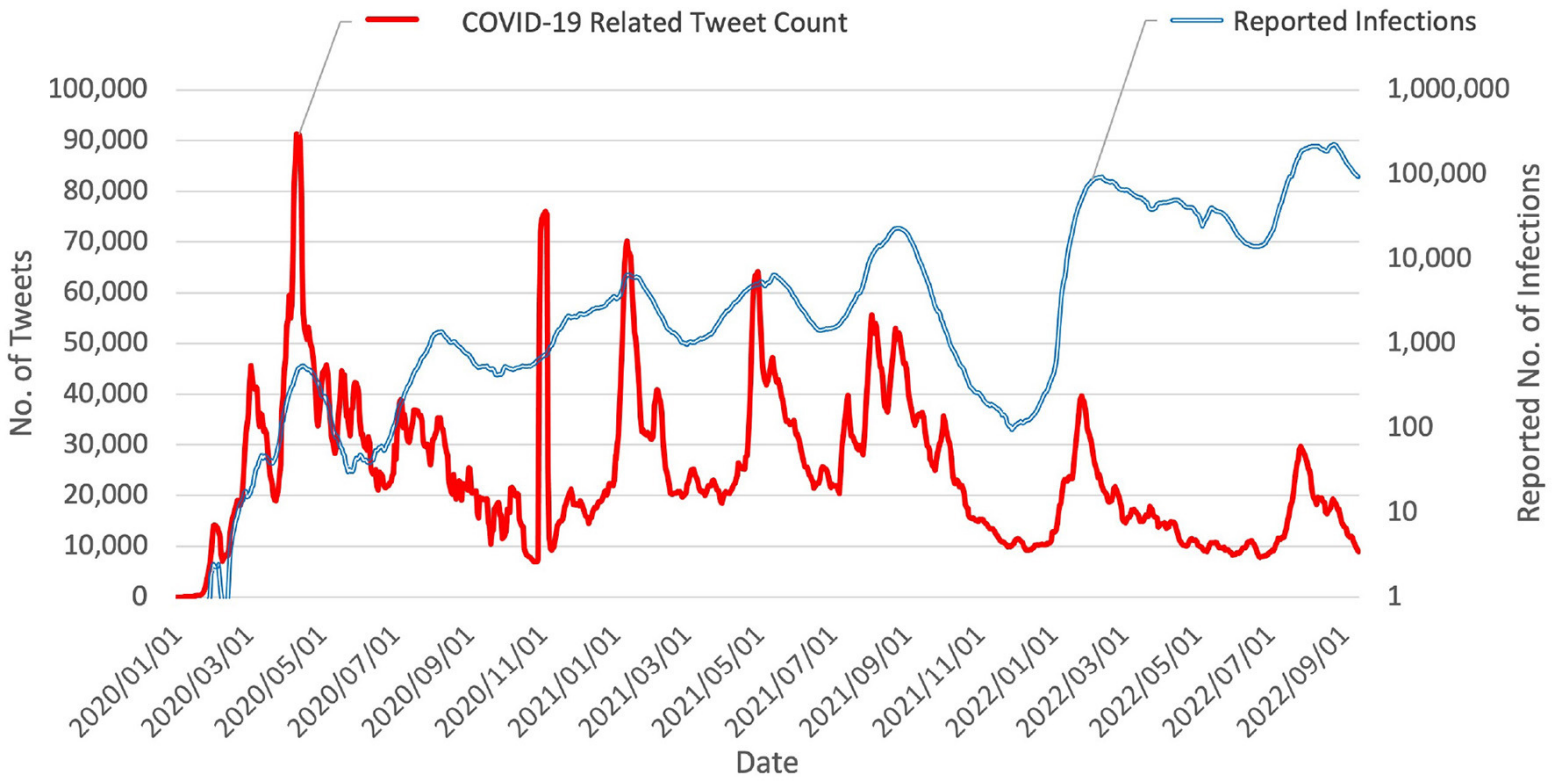
Chart of daily tweet count vs. reported number of COVID-19 infections in Japan (values were smoothed using 7-day moving average). Data suggest that number of COVID-19 related tweets has been correlated to some degree with progression of the epidemic in Japan since its beginning. An abnormal surge of social media reaction was observed on 29 and 30 October 2020, which is close to Halloween. Otherwise, the social media reaction exhibits waves corresponding to the seven waves of COVID-19 epidemic in Japan.

Figure 2 reveals a repetitive phenomenon: the reactions on Twitter form a wave shape corresponding to each wave of COVID-19. Tran and Matsui (27) hypothesized such a phenomenon on the basis of behavioral changes observed from Apple mobility trends reports. This phenomenon is also potentially due to excessive negative information exposure (13, 14) and heightened risk perception of social media users.

We performed a preliminary cross-correlation analysis between the COVID-19 related tweet count for each emoji and the reported number of COVID-19 cases over consecutive overlapping 28-day windows from 2020/01/01 to 2022/12/31 and examined the highest correlation and its corresponding lag value for each window. We found there existed a correlation (mean: 0.5920, standard deviation: 0.2518) and a considerable variance of lags with mean of –0.44 days and standard deviation of 6.06 days. Over all the windows, there are 25.64% of windows with positive lag values, 37.31% of windows with zero lag values, and 37.05% of windows with negative lag values. Details of the lag values for each emoji are shown in the Supplementary material. The existence of correlation with considerable variance of lag, even with the top popular emoji, shows that capturing long-term relationship between the social media emotion reaction and the COVID-19 epidemic could be difficult using linear models, a method capable of capturing non-linear long-term relationship could be promising. For this reason, we adopt LSTM which will be introduced later in Section 2.4.

To compare to countries other than Japan, we collect corresponding data from South Korea, Thailand, Indonesia, India, and Germany. The COVID-19 cases data are from WHO.^4^ Tweet counts of COVID-19 related tweets containing emoji are also collected using Twitter API. For these countries, instead of using self-designed keywords, we select tweets annotated with “COVID-19” domain entity^5^ by “context annotation” feature of Twitter API to select COVID-19 related tweets. Twitter only disclose limited details ^6^ that don't include specification of keywords used to do so. We also collect tweet count for Japan using the “context annotation” feature for comparison purpose. Due to privacy issues and, thus, Twitter location sharing “off” by default,^7^ the vast majority of Twitter users have their location sharing features off, so we rely on the most popular spoken language for collecting tweets from these countries^8^ The selected countries both have their distinguished languages popularly spoken in their own population and rank top in Twitter usage^9^. The languages are in the top 20 by tweet counts from Twitter Sample Stream API. Even though those countries also have other languages, those languages are minor or spoken by other countries, for example, Bengal (ranks 23^*rd*^ by the tweet counts) spoken by both India and Bangladesh (major language). Including those secondary languages risk including an overwhelming number of tweets from unexpected countries, so we chose to include only the primary language for those selected countries. The reaction with emoji is different among the 6 countries. The ratios of tweets containing emoji from 2020/01/01 to 2022/12/31 for each country are 4.08% (Germany), 8.27% (India), 8.02% (Indonesia), 8.66% (Japan), 2.84% (South Korea), and 4.60% (Thailand). The ratios are quite stable through out the period. Plots of the ratios are shown in the Supplementary material.

### 2.2. Estimation of the long-term tendency of social media engagement in Twitter

To identify the differences in social media reaction between COVID-19 waves, we calculated the total tweet count for a period of 84 days (12 weeks): 7 weeks before, 1 week during, and 4 weeks after each peak.

(1) $$\begin{array}{l} {\text{Total-Count}}_{x}=\underset{t}{\sum }x_{t} \end{array}$$

We also compared the total tweet count of the COVID-19 related tweet collection with that of the “all tweet” collection and calculated the ratio:

$$\frac{{\text{Total-Count}}_{\text{COVID-19 Related Tweets}}}{{\text{Total-Count}}_{\text{All Tweets}}}$$

### 2.3. Social media graph and anomaly detection

At certain moments in time, unexpected events occur that catch the attention of social media users. They become viral and spread rapidly over social media, leading to anomalous behavioral changes. Anomaly detection in social media has attracted attention from the research community (28), and several research efforts have demonstrated different findings representing the characteristics of social media evolution. Several anomaly types in social media have been studied including anomalous nodes, anomalous edges, anomalous sub-graphs, and anomalous events.

Intuitively, the state of reactions on social media can be represented as a graph that connects social media objects including users, posts, entities, and topics. The graph can then be used to analyze behavioral evolution. The graph continues to evolve as new users join in, new posts are shared, new topics are discussed, and new entities are mentioned.

In the work of Rossi et al. (29), large time-evolving graphs were analyzed for anomaly detection. They found that it is possible to identify interesting patterns and detect unusual structural transitions. In a large Twitter relationships network, they observed seasonality among the transitions. In particular, they found that users generally behave much differently over the weekends, as evidenced by an increase in the anomalous scores on those days. They speculated that the manner of tweeting differs between weekends and workdays. Motivated by their findings, we adapted their method for use in analyzing our Twitter graph, looking for clues to the factors that trigger anomalous behavioral changes on Twitter.

We performed the analysis using an evolving dynamic Twitter graph. We focused on capturing the temporal behavioral changes in interconnected social media objects (users, emoji, hashtags, domains, and entities) on the social media platform as it evolved during the COVID-19 epidemic in Japan. The social media were represented as a heterogeneous graph connecting the social media objects.

In this section, we introduce our approach utilizing a graph to represent Twitter data and our method for identifying the anomalous temporal behavioral changes in the Twitter objects. We expected that the social media network structural changes identified with our approach would complement the use of the tweet count to enhance our COVID-19 case prediction system.

#### 2.3.1. Twitter graph

To construct our Twitter graph, we considered five Twitter objects: user, emoji, hashtag, entity, and domain. These objects are connected to the event of a tweet being posted, commented upon, or retweeted (shared) and comprise the nodes of the graph.

- User: Twitter member.
- Hashtag: a way for users to include their tweets into a (trending) broad topic of conversation.
- Entity: named entity; for instance, person, organization, location, time, which is automatically annotated by Twitter's named-entity recognition system given the user tweet's full-text.
- Domain: domain context of tweet as defined by Twitter.
- Emoji: graphical representations of emotions, e.g., 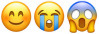.

The graph also has five types of edges (relations):

- User → User: A user mentions another user in a tweet replying to the mentioned user's tweet or for tagging an additional user into the current conversation.
- User → Hashtag: A user posts or shares a tweet containing a hashtag. The user wants to include their post into a certain broader topic of conversation.
- User → Emoji: A user posts or shares a tweet containing an emoji. The user wants to express a certain emotion.
- User → Domain: A user posts or shares a tweet belonging to a domain.
- User → Entity: A user posts or shares a tweet mentioning an entity.

Formally, we define a graph *G* = (*N, E*), where *N* is the set of nodes and *E* is the set of edges in *G*. At time slice *t*, snapshot *G*_*t*_ = (*N*_*t*_, *E*_*t*_) is a subgraph of *G* with active edges *E*_*t*_ connecting active nodes *N*_*t*_. For smoothly capturing graph state transitions instead of capturing the graph at separate and short time points, we use moving and overlapping time slices for taking snapshots of *G*. For instance, if the time slice is 7 days, snapshot *G*_*t*_ is constructed with active nodes and edges over days [*t* − 6, *t*].

#### 2.3.2. Graph feature representation

Following the work of Rossi et al. (29), we estimate the latent features, which are called the “roles of the graph," and use them to describe the behaviors of the graph. A role transition model is used to capture the behavioral transitions of the nodes in each snapshot over time *t*.

**Features**. Two categories of features are considered: basic features and recursive features. In accordance with the definitions of Henderson et al. (30), the basic features are node degree, weight, and egonet measure, taking into account in-coming and out-going directions. The recursive features are aggregations of the basic features and previously discovered recursive features using sum and/or mean. We also applying feature pruning using logarithmic binning (30). Formally, we denote *V* = {*V*_*t*_} as the features obtained for snapshot {*G*_*t*_}.

**Roles**. By applying latent semantic analysis using non-negative matrix factorization (NMF), we find the latent feature space considered to be the role of each node. Nodes with similar role representations can be considered to be in one group with a common role in the graph. We estimate the role representations as a low-rank *r* matrix $R_{t}\in {\mathbb{R}}^{n\times r}$ of the nodes of snapshot *G*_*t*_ as *R*_*t*_*F* ≈ *V*_*t*_ using NMF for reasons of interpretability and efficiency (29). The value of *r* is chosen such that *r* < *min*_*t*_(*n*_*t*_, *f*), where *n*_*t*_ is the number of active nodes at time *t*, and *f* is the number of discovered features. In total, we obtained *R* = {*R*_*t*_} as the role representations for all snapshots of *G*.

**Role transition model**. The estimated role transition can be used to analyze how the graph evolves over time. Given the high interpretability of NMF, we used it to estimate the role transitions. We estimate a transition matrix *T* such that *R*_*t*−1_*T* ≈ *R*_*t*_.

#### 2.3.3. Anomaly analysis

The idea of anomaly analysis is that, if the prediction of the next role of a node diverges from the observation, the divergence value represents the anomalous score (29). The higher the divergence, the more abnormal the behavioral change of the node when interacting with the other nodes in the graph. Given a role transition matrix *T* estimated using

(2) $$\begin{array}{l} R_{t-k-1}T\approx R_{t-k}\text{for}\forall k\in \left[1,K\right], \end{array}$$

we can predict the next role representation at time *t* as

(3) $$\begin{array}{l} {\hat{R}}_{t}=R_{t-1}T. \end{array}$$

The divergence of the predicted ${\hat{R}}_{t}$ from the observed *R*_*t*_ is considered to be the anomaly and can be measured as

(4) $$\begin{array}{l} ||{\hat{R}}_{t}-R_{t}|{|}_{F}, \end{array}$$

Where || · ||_*F*_ is the Frobenius norm. In this study, we set *K* = 14 days.

In proceeding to the next step of building our prediction system, we need to obtain anomalous scores for the emoji. For a given emoji identified as node *i* of the graph, we obtain the emoji's anomalous score as the divergence of the predicted role vector ${\hat{R}}_{t}^{\left(i\right)}$ (row *i*^*th*^ of ${\hat{R}}_{t}$) from the observed role vector $R_{t}^{\left(i\right)}$:

(5) $$\begin{array}{l} \left|{\hat{R}}_{t}^{\left(i\right)}-R_{t}^{\left(i\right)}\right| \end{array}$$

The role transition matrix *T* captures the global transition of the graph in the period of *K* days. The predicted role matrix ${\hat{R}}_{t}$ is, therefore, the expected next role representation by the global transition. Hence, the anomalous score computed by Equation 5 represents the anomaly of the node as how it diverges from the global transition.

### 2.4. COVID-19 case prediction system

Studies of the effects of social media on societal events including COVID-19 have led to social media information being used for constructing COVID-19 case prediction systems. In this study, we investigated the effectiveness of using emoji, which are commonly used in social media communications, in the prediction of COVID-19 cases.

**Figure 3:**
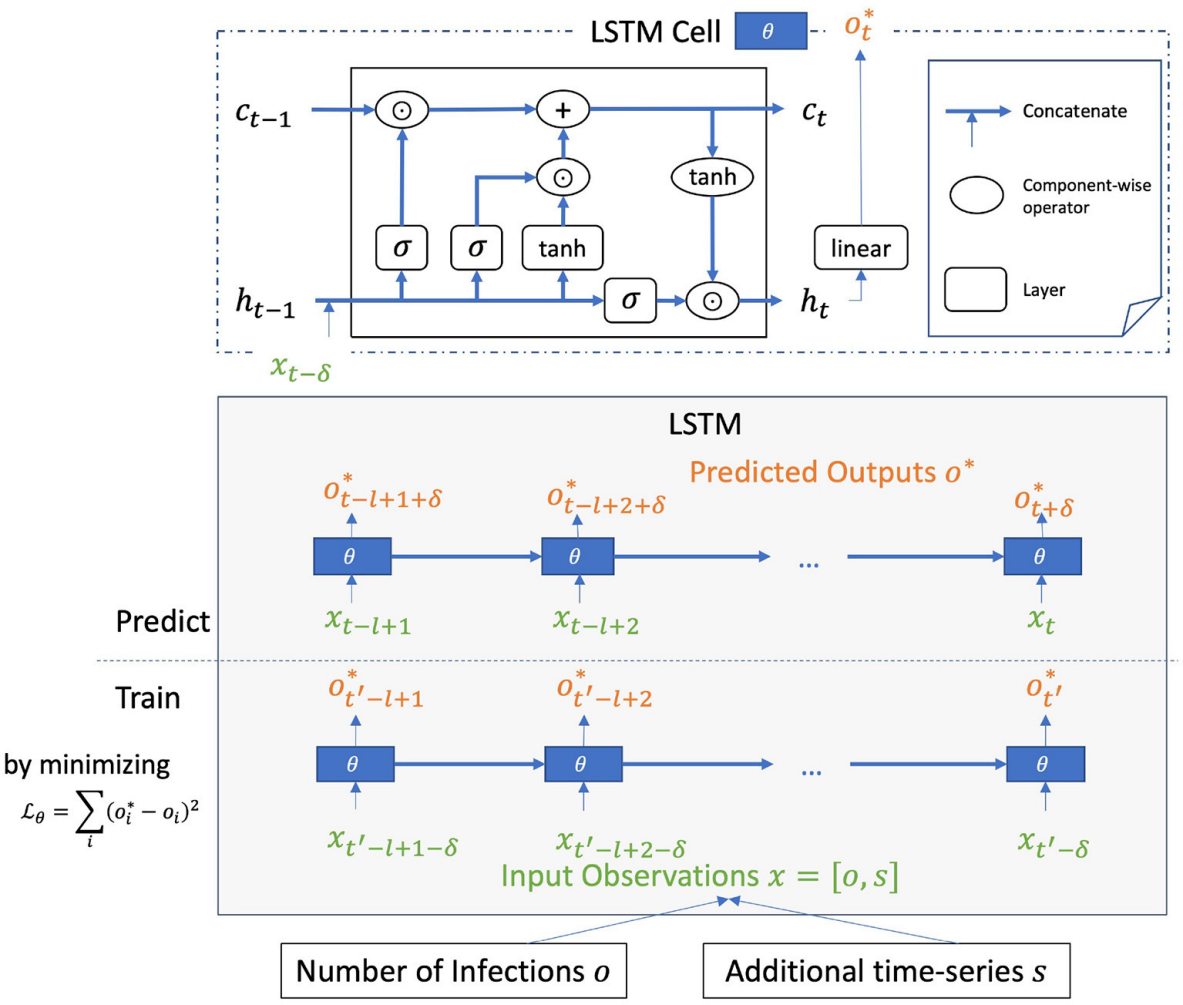
LSTM model for COVID-19 case prediction with time lag δ configured to work on memory length of *l*. In inference step, with input sequence {*x*_*t*−*l*+1_, …, *x*_*t*_} of length *l*, the model predicts the number of COVID-19 cases $o_{t+\delta }^{*}$, i.e., at δ time steps since the last input time step. Additional time-series *s* is for one of the emoji features (tweet count or anomalous score).

**A system of multiple LSTM models**. Our COVID-19 case prediction system is constructed using an ensemble of long short-term memory (LSTM) models. LSTM was proposed by Hochreiter and Schmidhuber (23) and is widely and successfully used in modeling sequential data (31). For each LSTM model in the ensemble, given an input sequence ${\bar{x}}_{t,l}=\left\{x_{t-l+1},...,x_{t}\right\}$ of length *l*, the model is trained to output the number of COVID-19 cases $o_{t+\delta }^{*}$, i.e., at δ time steps since the last input time step. The input contains the observed number of COVID-19 cases *o* and an additional feature *s*, which is either the tweet count or the anomalous score of an emoji. Each LSTM model is configured with 4 layers and hidden size of 16 each layer. The operation of an LSTM cell, the building block of the LSTM model illustrated in Figure 3 can be described in the following Equations (6–12), where the vanilla LSTM cell is extended with a linear layer to map the high-dimensional hidden state *h*_*t*_ to a single-value prediction $o_{t}^{*}$.

(6) $$\begin{array}{l} i_{t}=\sigma \left(W_{ii}x_{t-\delta }+b_{ii}+W_{hi}h_{t-1}+b_{hi}\right) \end{array}$$

(7) $$\begin{array}{l} f_{t}=\sigma \left(W_{if}x_{t-\delta }+b_{if}+W_{hf}h_{t-1}+b_{hf}\right) \end{array}$$

(8) $$\begin{array}{l} g_{t}=\text{tanh}\left(W_{ig}x_{t-\delta }+b_{ig}+W_{hg}h_{t-1}+b_{hg}\right) \end{array}$$

(9) $$\begin{array}{l} j_{t}=\sigma \left(W_{ij}x_{t-\delta }+b_{ij}+W_{hj}h_{t-1}+b_{hj}\right) \end{array}$$

(10) $$\begin{array}{l} c_{t}=f_{t}⊙c_{t-1}+i_{t}⊙g_{t} \end{array}$$

(11) $$\begin{array}{l} h_{t}=j_{t}⊙\text{tanh}\left(c_{t}\right) \end{array}$$

(12) $$\begin{array}{l} o_{t}^{*}=W_{o}h_{t}+b_{o} \end{array}$$

The gating mechanism, a specialized feature of LSTM, with input gate *i*_*t*_, forget gate *f*_*t*_, and output gate *j*_*t*_ controls the information flow, and, thus, helps learn important long term memory captured in the cell state *c*_*t*_, which is intuitively beneficial for learning long term dependency between social media reaction and epidemic situation.

**A multi-feature ensemble**. Using the historical number of cases and one additional feature as input, we construct a prediction system that is an ensemble of LSTM models of different emoji features {emoji} × {tweet count and anomalous score}. Instead of constructing a complex model with the inputs being the number of COVID-19 cases and many additional features, we construct an ensemble of simple models, with each one focused on modeling the relationship between the number of COVID-19 cases and one predictor or one emoji feature.

**A dynamic ensemble of the best models**. Only the best models are selected to be used in the ensemble at a certain time step. We consider that a model is better than the others if its performance was better at the most recent time step. Let *t* = *t*_0_ + Δ*t* be the time step at which we want to make prediction *y*_*t*_ given input ${\bar{x}}_{t_{0},l}=\left\{x_{t_{0}-l+1},...,x_{t_{0}}\right\}$, where *t*_0_ represents the last input time step. From all the trained models {*f*}, the best model $f_{\delta }^{*,1}$ for time lag δ at time *t* is selected on the basis of

(13) $$\begin{array}{l} f_{\delta }^{*,1}={\text{argmin}}_{f_{\delta }}\left\{\frac{1}{\tau_{2}-\tau_{1}+1}\overset{\tau_{2}}{\underset{k=\tau_{1}}{\sum }}\text{ERR}\left(f_{\delta }\left({\bar{x}}_{t-k-\delta ,l}\right),{ŷ}_{t-k}\right)\right\}, \end{array}$$

(14) $$\begin{array}{l} \text{where }{ŷ}_{t-k}=\{\begin{array}{ll} y_{t-k} & \text{if }t-k>t_{0}\\ o_{t-k} & \text{otherwise} \end{array}, \end{array}$$

and ERR(·, ·) is the mean relative absolute error described in Equation (17). The 2^*nd*^, …, *m*^*th*^ best models $f_{\delta }^{*,2},...,f_{\delta }^{*,m}$ are also selected. The prediction at time *t* = *t*_0_ + Δ*t* is given by

(15) $$\begin{array}{l} y_{t=t_{0}+\Delta t}=\frac{1}{\hat{\delta }}\frac{1}{m}\overset{\hat{\delta }+\Delta t-1}{\underset{\delta =\Delta t}{\sum }}\overset{m}{\underset{i=1}{\sum }}f_{\delta }^{*,i}\left({\bar{x}}_{t-\delta ,l}\right). \end{array}$$

The parameter $\hat{\delta }$ enables the smoothness of the prediction to be controlled. The higher the value of $\hat{\delta }$, the longer the period of recent events that the system takes into account, resulting in smoother prediction. The lower the value of $\hat{\delta }$, the more sensitive the prediction is to the most recent events. For each emoji feature, a set of *r* models is trained with different randomly initialized parameters, which results in the total number of trained models being |{*f*}| = |{emoji} × {tweet count, anomalous score}| × *r*. This enables models trained for the same emoji feature to be selected and used in the ensemble if they perform well with different sets of weights. We employed these dynamic ensemble parameters: τ_1_ = 1, τ_2_ = 7, *m* = 10, and $\hat{\delta }=1$.

**Data Smoothing**. Inputs including the number of cases and additional time series (emoji usage count, emoji anomalous score) are smoothed using a 7-day moving average. The predicted number of cases is therefore a 7-day moving average. We consider the 7-day moving average smoothing as an appropriate way to have stable analysis of the data and a mitigation of case fluctuations due to reasons including human errors, issues in local municipality's reporting mechanism, and the going-to-test timing of residents.

**Training**. The system is trained or updated by utilizing data assimilation so that previously trained models are updated when additional observations are available. For example, if time lag δ = 1, when the data at time *t* are observed, the model trained up to time *t* − 1 is updated or tuned using the additional data observed at time *t*. Memory length *l* is set on the basis of the corresponding time lag δ:

(16) $$\begin{array}{l} l=\left\lfloor \frac{3}{2}\delta +14\right\rfloor , \end{array}$$

Where ⌊·⌋ is the round down operator. Memory length *l* is calculated such that, the further into the future the system has to predict, the further into the past the system needs to look. The data until 2020/09/07 (before the 3^*rd*^ wave) were used for obtaining initial models with a maximum of 10,000 training epochs and early stopping using 5% of the data held out as development set, before the data assimilation stage. Each data assimilation run is carried out with 25 fine-tuning epochs.

**Evaluation**. The prediction error is measured by the mean relative absolute error (MRAE):

(17) $$\begin{array}{l} \text{ERR}\left(y,o\right)=\frac{1}{n}\overset{n}{\underset{i=1}{\sum }}\frac{\left|y_{t+i}-o_{t+i}\right|}{\left|o_{t+i}\right|}, \end{array}$$

Where *y, o* ∈ ℝ^*n*^ are the system prediction and ground truth, respectively, and *n* is the size of the evaluation window {*t* + 1, …, *t* + *n*}, where the system predicts {*y*_*t*+1_, …, *y*_*t*+*n*_} given the input data up to *x*_*t*_. As we perform analysis for a long period where the domain of values for the COVID-19 cases changed dramatically through the course of waves, we select MRAE as the evaluation metric because of its popular adoption in time-series forecasting studies and its advantage of evaluating outputs with large value fluctuation. Given an evaluation period *d*, and an evaluation window size *n* ≤ *d*, we compute an error value for each of the *d* − *n* + 1 consecutive overlapping evaluation windows using Equation (17). Then, in the later sections, we will report system performance with the **mean** and **standard deviation** of the errors for the evaluation windows, and illustrate system comparison with “**better error x% of the time**” indicating the number of evaluation windows, in percentage, where the preferred system *S*_*pref*_ achieves better error than the referenced system *S*_*ref*_ (Equation 18).

(18) $$\begin{array}{l} \text{better error x\% of the time}\left(S_{pref},S_{ref},d,n\right)\\ =\frac{\sum_{w=1}^{d-n+1}1_{ERR\left[S_{pref},w\right]<ERR\left[S_{ref},w\right]}}{d-n+1}\times 100\% \end{array}$$

## 3. Results of Japan

### 3.1. Long-term trend of social media reaction on Twitter

**Figure 4:**
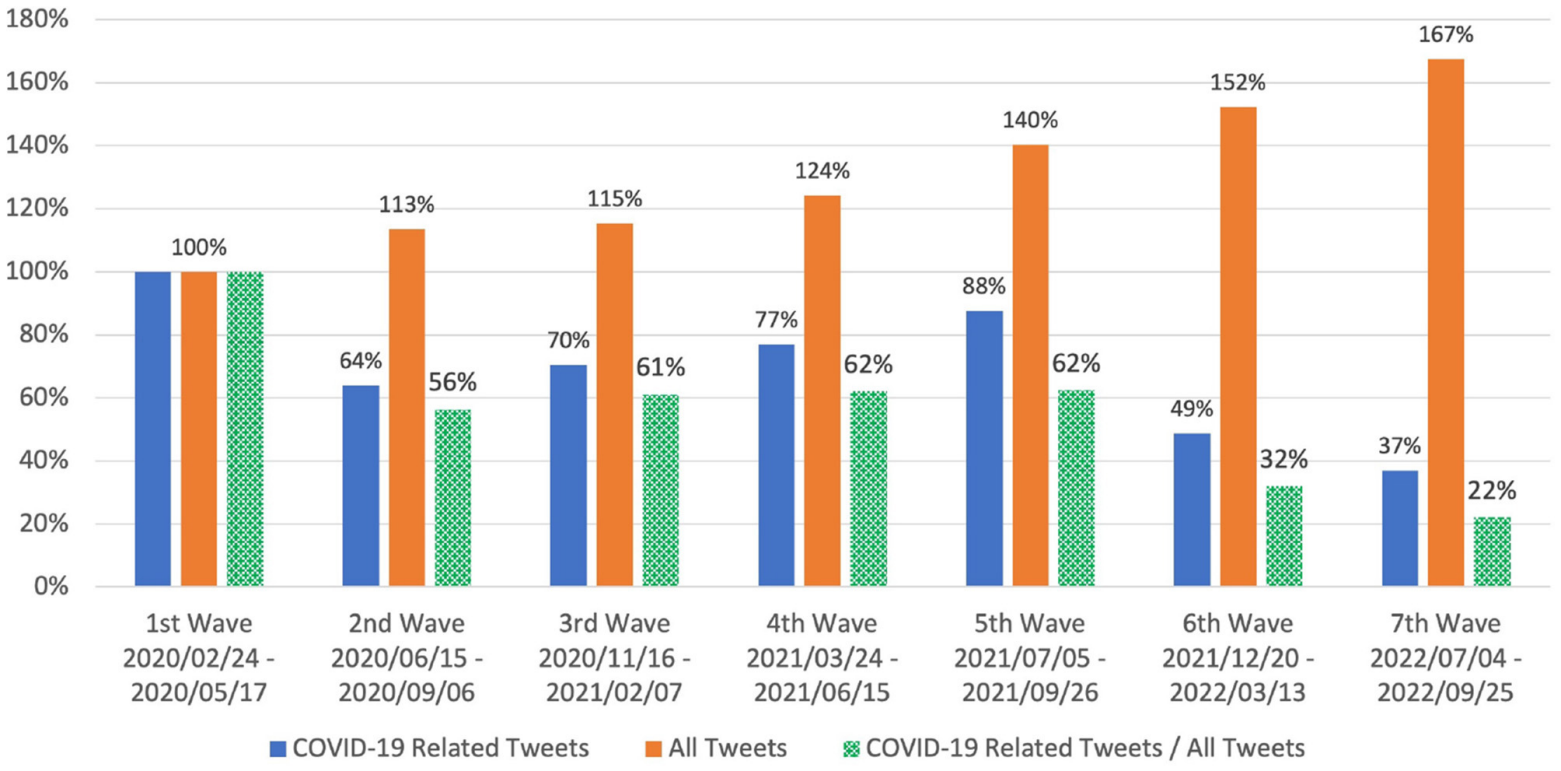
Social media (Twitter) reaction in Japan measured by total tweet count (Equation 1) for each COVID-19 wave over period of 12 weeks: 7 weeks before, 1 week during, and 4 weeks after peak of corresponding wave. Values shown are relative to those for 1st wave. Social media reaction in 6th and 7th waves was obviously much less than that in previous waves. Additionally, total tweet count ratio (“COVID-19 related tweets” vs. “all tweets”) dropped to 32% in 6th wave and 22% in 7th wave compared with 56-62% in 2nd - 5th waves.

From Figure 4, it can be easily seen that the social media reaction in the subsequent waves (2nd – 7th) was noticeably reduced compared with that in the 1st wave. It was particularly smaller in the 6th and 7th waves. Despite social media reaction in general increasing by 167% in the 7th wave compared with that in the 1st wave, the attention on COVID-19 dropped to 37% from 64 to 88% in the 2nd - 5th waves and most recently 49% in the 6th wave. This led to the total tweet count ratio of “COVID-19 related tweets” to “all tweets” (the “COVID-19 attention proportion”) falling to 32% in the 6th wave and 22% in the 7th wave compared with 56-62% in the 2nd – 5th waves. Overall, social media attention on COVID-19 vs. general topics on Twitter dropped by 38–44% from the 1st wave to the 5th wave and by 68–78% from the 1st wave to the 6th and 7th waves.

### 3.2. Anomaly detection analysis

**Figure 5:**
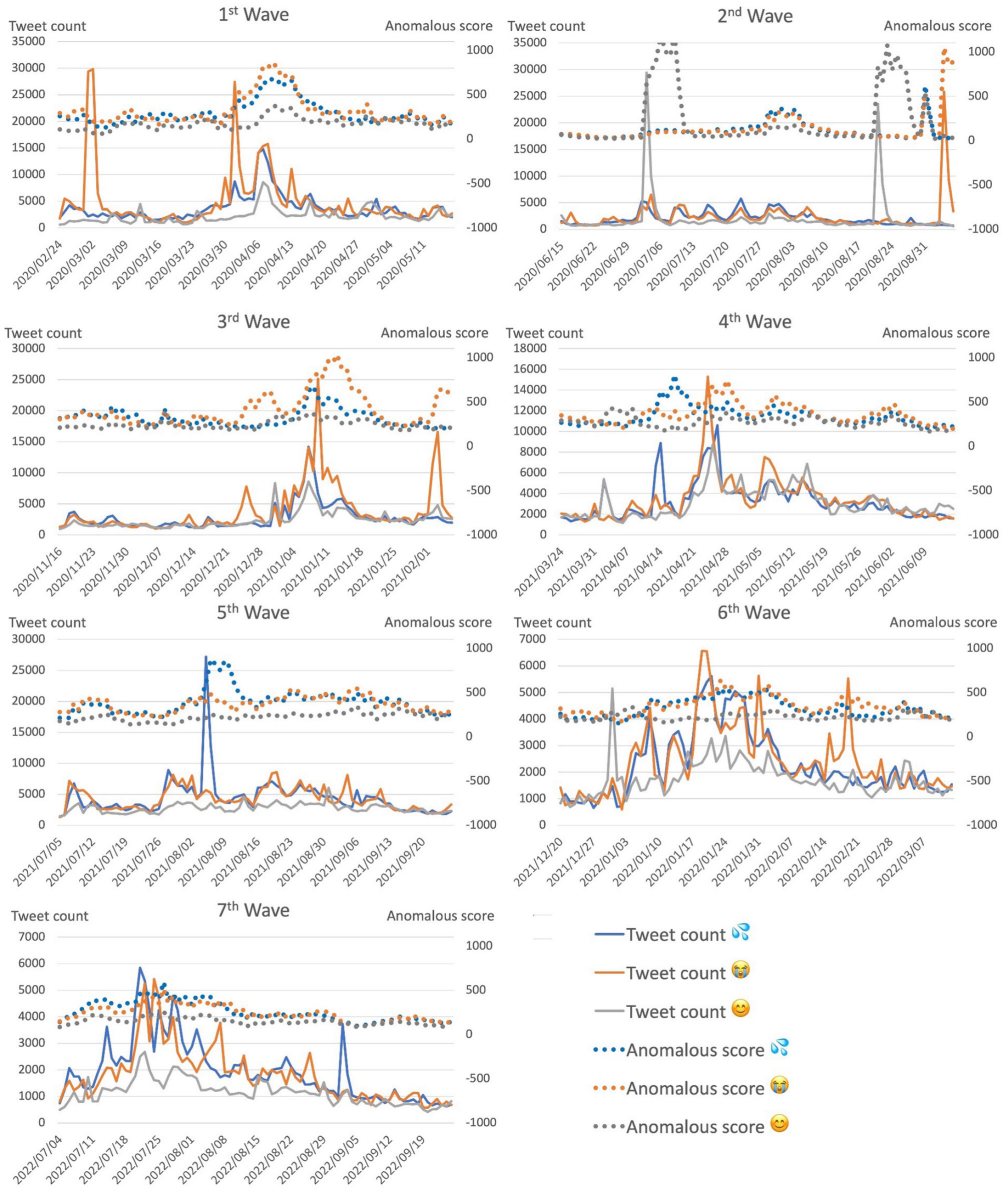
Anomalous scores of top-3 used emoji with corresponding tweet counts for each COVID-19 wave over period of 12 weeks: 7 weeks before, 1 week during, and 4 weeks after peak of corresponding wave. Anomalous scores represent situations in which there were surges in social media reaction, which resulted in structural changes in social media network.

As illustrated in Figure 5, the anomalous score aligned with the tweet count most of the time. This suggests that surges in social media reaction are accompanied by structural changes in social media networks. This was particularly the case for periods leading up to a wave peak, where we observed a surge in both tweet count and anomalous score. This means that, in addition to using the tweet count to capture the surface trend of social media reaction, we can also capture the magnitude of the structural changes in the social media network during such periods.

However, there were several situations in which social media reaction surged while the anomalous score did not follow, for instance, around 2020/03/02 and 2022/09/05. Furthermore, for the top 3 emoji (Figure 5), around 2022/02/21, the anomalous score for the crying face emoji was similar to that for the other two emoji, sweating and smiley, and did not align with the corresponding tweet count. Such rare occurrences would, however, have little effect on system performance due to the design of the prediction system as an ensemble of independent models.

### 3.3. COVID-19 case prediction

**Figure 6:**
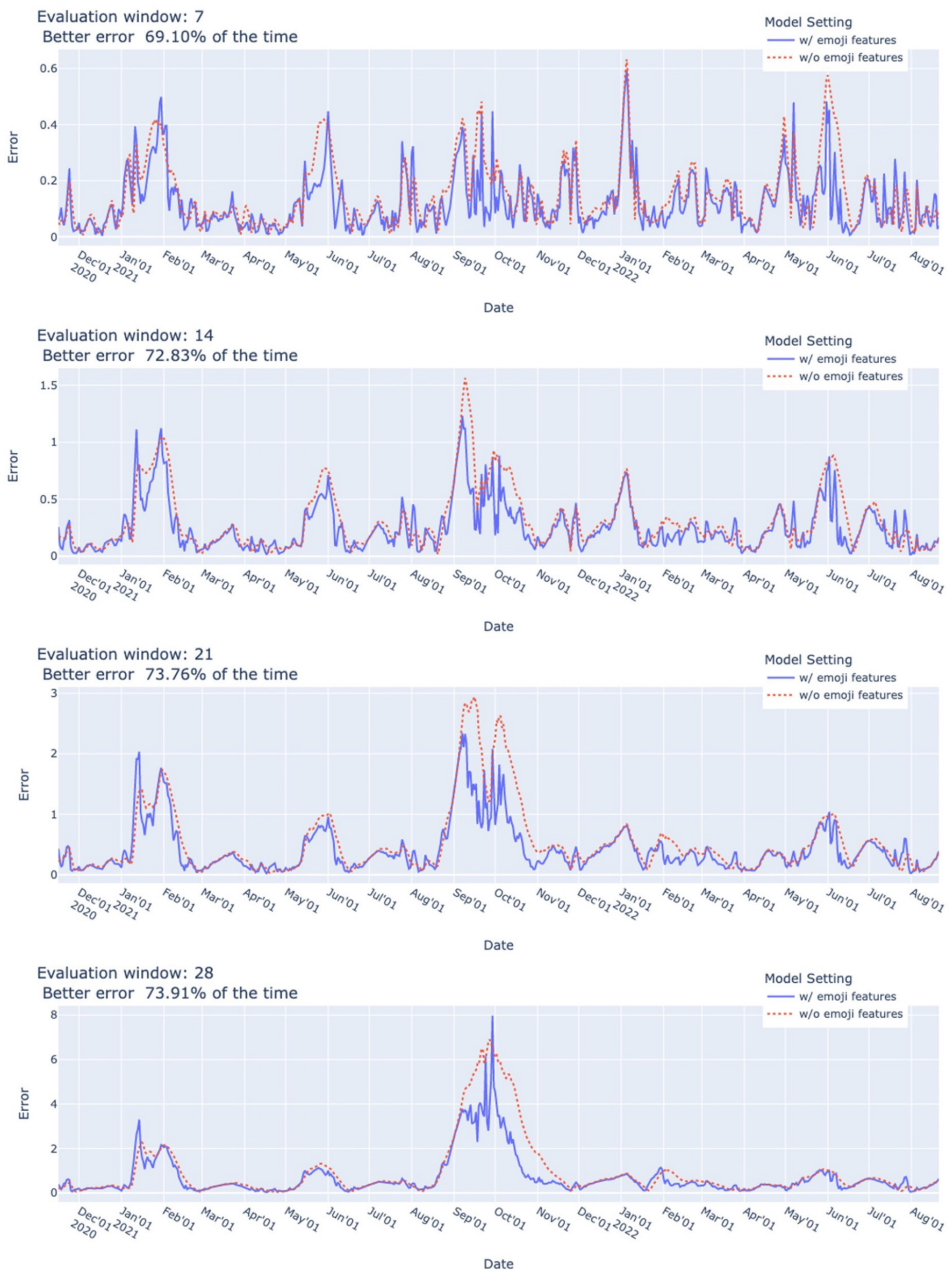
COVID-19 case prediction system performance (MRAE). Error at given date was measured on basis of prediction for evaluation window starting from given date from 2020/11/16 to 2022/08/21. Using additional emoji features yielded better error 69.10–73.91% of the time. Relative error reduction ranged from 0.10 to 94.14% with mean of 28.77% and median of 23.91%. *Dynamic ensemble parameters*: $\tau_{1}=1,\tau_{2}=7,m=10,\hat{\delta }=1$.

Our experimental results show that using additional emoji features improves prediction performance in terms of MRAE. As shown in Figure 6, in the evaluation period from 2020/11/16 to 2022/08/21 (+6, +13, +20, or +27 days depending on the evaluation window), using additional emoji features achieved better error 69.10–73.91% of the time. The improvement in term of relative error reduction ranged from 0.1 to 94.14% with a mean of 28.77% and median of 23.91%. As shown in Table 2, improvement was evident most of the time during the 7 weeks before the week of each wave.

**Table 2:** COVID-19 case prediction system performance with and without use of emoji for each wave.

**Wave**	**Window starting date**	**Window = 7**	**Window = 14**	**Window = 21**	**Window = 28**
**With emoji features**					
3rd wave	2020/11/16–2021/01/03	6.39(4.90)	**11.23**(7.86)	**18.29**(10.65)	24.39(11.73)
4th wave	2021/03/22–2021/05/09	**4.98**(3.40)	**9.69**(5.73)	**13.82**(8.18)	**17.99**(10.79)
5th wave	2021/07/05–2021/08/22	**10.25**(8.06)	**20.10**(11.11)	**28.20**(12.67)	**40.19**(16.62)
6th wave	2021/12/20–2022/02/06	**15.91**(14.25)	**28.77**(18.95)	**44.98**(19.15)	**61.47**(23.53)
7th wave	2022/07/04–2022/08/21	9.15(6.36)	**16.00**(11.12)	**25.50**(15.97)	**35.41**(17.77)
**Without emoji features**					
3rd wave	2020/11/16–2021/01/03	**6.22**(4.15)	12.18(5.86)	19.63(8.86)	**24.37**(8.71)
4th wave	2021/03/22–2021/05/09	5.69(2.91)	10.79(6.21)	14.88(10.50)	21.11(13.21)
5th wave	2021/07/05–2021/08/22	10.49(6.84)	21.18(8.82)	29.38(12.30)	41.43(16.76)
6th wave	2021/12/20–2022/02/06	17.03(14.66)	33.27(18.20)	51.09(19.46)	64.21(25.43)
7th wave	2022/07/04–2022/08/21	**8.66**(4.79)	16.68(11.80)	26.18(16.62)	36.07(18.15)

The values are mean and standard deviation (in parentheses) of MRAE of consecutive overlapping evaluation windows with starting date 7 weeks before week of corresponding peak. Performance was generally better when emoji features were used. The error values are shown in percentage with % omitted. The bold values indicate better performance.

As also shown in Table 2, the system performed better for the 4th wave compared with the 3rd wave, worse for the 5th wave, and the worst for the 6th wave. From another perspective, significant situational differences were observed for the 5th and the 6th waves. The 5th wave was characterized by the COVID-19 Delta variant, a significantly more fatal variant. The 6th wave was characterized by the Omicron variant, a much more infectious variant, but less deadly. Therefore, there was no declaration of an emergency for this wave.

The results of an experiment on system performance with the use of tweet count and anomalous score together and alone for each wave (Table 3) show that using both improved system performance in terms of the MRAE. Out of the 20 evaluations, the combination yielded improvement ten times and equal performance once, tweet count yielded better performance only eight times, and anomalous score yielded better or equal performance only twice.

**Table 3:** COVID-19 case prediction system performance with use of tweet count and anomalous score together and alone for each wave.

**Wave**	**Window starting date**	**Window = 7**	**Window = 14**	**Window = 21**	**Window = 28**
**Both tweet count and anomalous score**					
3rd wave	2020/11/16–2021/01/03	**6.39**(4.90)	**11.23**(7.86)	**18.29**(10.65)	**24.39**(11.73)
4th wave	2021/03/22–2021/05/09	**4.98**(3.40)	**9.69**(5.73)	13.82(8.18)	17.99(10.79)
5th wave	2021/07/05–2021/08/22	10.25(8.06)	20.10(11.11)	28.20(12.67)	**40.19**(16.62)
6th wave	2021/12/20–2022/02/06	**15.91**(14.25)	**28.77**(18.95)	**44.98**(19.15)	**61.47**(23.53)
7th wave	2022/07/04–2022/08/21	9.15(6.36)	16.00(11.12)	25.50(15.97)	35.41(17.77)
**Anomalous score only**					
3rd wave	2020/11/16–2021/01/03	6.44(5.15)	11.45(7.77)	18.57(10.53)	24.45(11.06)
4th wave	2021/03/22–2021/05/09	**4.98**(3.26)	9.71(5.53)	14.05(8.22)	18.46(11.06)
5th wave	2021/07/05–2021/08/22	10.15(7.31)	20.70(10.17)	29.16(11.64)	41.19(16.89)
6th wave	2021/12/20–2022/02/06	16.21(14.19)	29.94(18.30)	46.61(17.97)	63.57(22.98)
7th wave	2022/07/04–2022/08/21	**9.06**(6.11)	16.21(11.81)	25.65(16.60)	35.43(18.35)
**Tweet count only**					
3rd wave	2020/11/16 - 2021/01/03	6.57(5.05)	11.68(8.27)	19.04(10.90)	24.97(11.99)
4th wave	2021/03/22–2021/05/09	5.11(3.21)	10.07(5.40)	**13.54**(8.31)	**17.70**(11.01)
5th wave	2021/07/05–2021/08/22	**9.86**(7.76)	**19.97**(10.74)	**28.14**(12.14)	40.26(16.55)
6th wave	2021/12/20–2022/02/06	16.22(14.48)	28.91(19.28)	45.19(19.36)	61.64(23.41)
7th wave	2022/07/04–2022/08/21	9.39(5.78)	**15.88**(11.09)	**25.42**(15.56)	**35.31**(17.00)
**Tweet count only (using context annotation)**					
3rd wave	2020/11/16–2021/01/03	6.42(4.96)	**10.69**(7.18)	**17.01**(7.76)	**23.01**(7.45)
4th wave	2021/03/22–2021/05/09	5.24(2.94)	10.34(5.20)	14.11(7.93)	**17.65**(10.62)
5th wave	2021/07/05–2021/08/22	10.31(8.00)	21.21(11.29)	29.47(11.37)	40.85(15.24)
6th wave	2021/12/20–2022/02/06	17.09(14.33)	29.08(19.27)	45.06(19.72)	61.48(23.40)
7th wave	2022/07/04–2022/08/21	**8.42**(5.92)	**15.85**(12.11)	**24.96**(16.48)	**34.53**(17.55)

The values are mean and standard deviation (in parentheses) of MRAE of consecutive overlapping evaluation windows with starting date 7 weeks before week of corresponding peak. Performance was generally better when both emoji feature categories were used. The error values are shown in percentage with % omitted. Decoration: **bold**+underline - the 4th setting gets the best result over the other 3 settings, underline - the 4th setting gets better result than the 3rd setting, **bold** - best in the first 3 settings.

**Figure 7:**
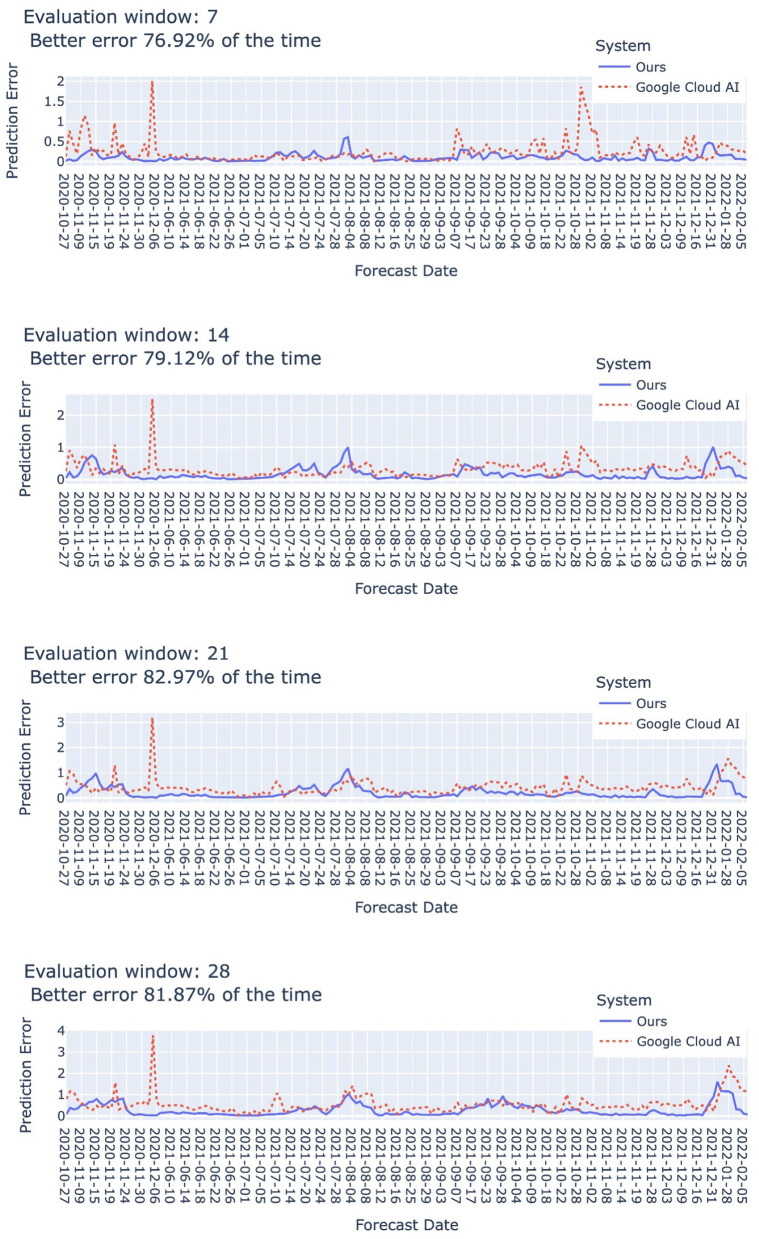
Comparison with Google Cloud AI (32). The “forecast date” indicates the date when each system is executed to produce the predicted number of cases for the next 7, 14, 21, and 28 days. All the periods indicated by the forecast dates are taken from the “COVID-19 Public Forecasts” dataset by Google Cloud AI. Our system achieved better error in 76.92–81.87% of the time. *Dynamic ensemble parameters*: $\tau_{1}=1,\tau_{2}=7,m=10,\hat{\delta }=1$.

Our system achieved competitive performance when comparing with Google Cloud AI forecasting system (32). As shown in Figure 7, in the forecast periods reported by Google Cloud AI forecasting system, our system could achieve better prediction error in 76.92–81.87% of the time. Their system was designed with consideration of a number of features including, for instance, per capita income, hospital patient experience rating, air quality measures, mobility index, and governmental policies such as restricting restaurants and school closure, but social media emotion was not considered. They published the “COVID-19 Public Forecasts”^10^ dataset containing the prediction outputs for U.S. and Japan.

## 4. Results of other countries

In this section, we present the results of our method for Germany, India, Indonesia, South Korea and Thailand. Like Japan, these countries are the tops in Twitter usage and have their tweets conveniently collected by their primary spoken languages. Location-based filtering is difficult since Twitter users are turning off location sharing as their concerns of privacy issue. Due to Twitter API capping the number of tweets that can be downloaded (10M/month^11^), we don't present the anomaly detection analysis for these countries in this paper. Tweet count data as mentioned in Section 2.1 are collected using Twitter API “context annotation” feature. We also put the results of Japan as comparison.

### 4.1. Social media reaction on Twitter

**Figure 8:**
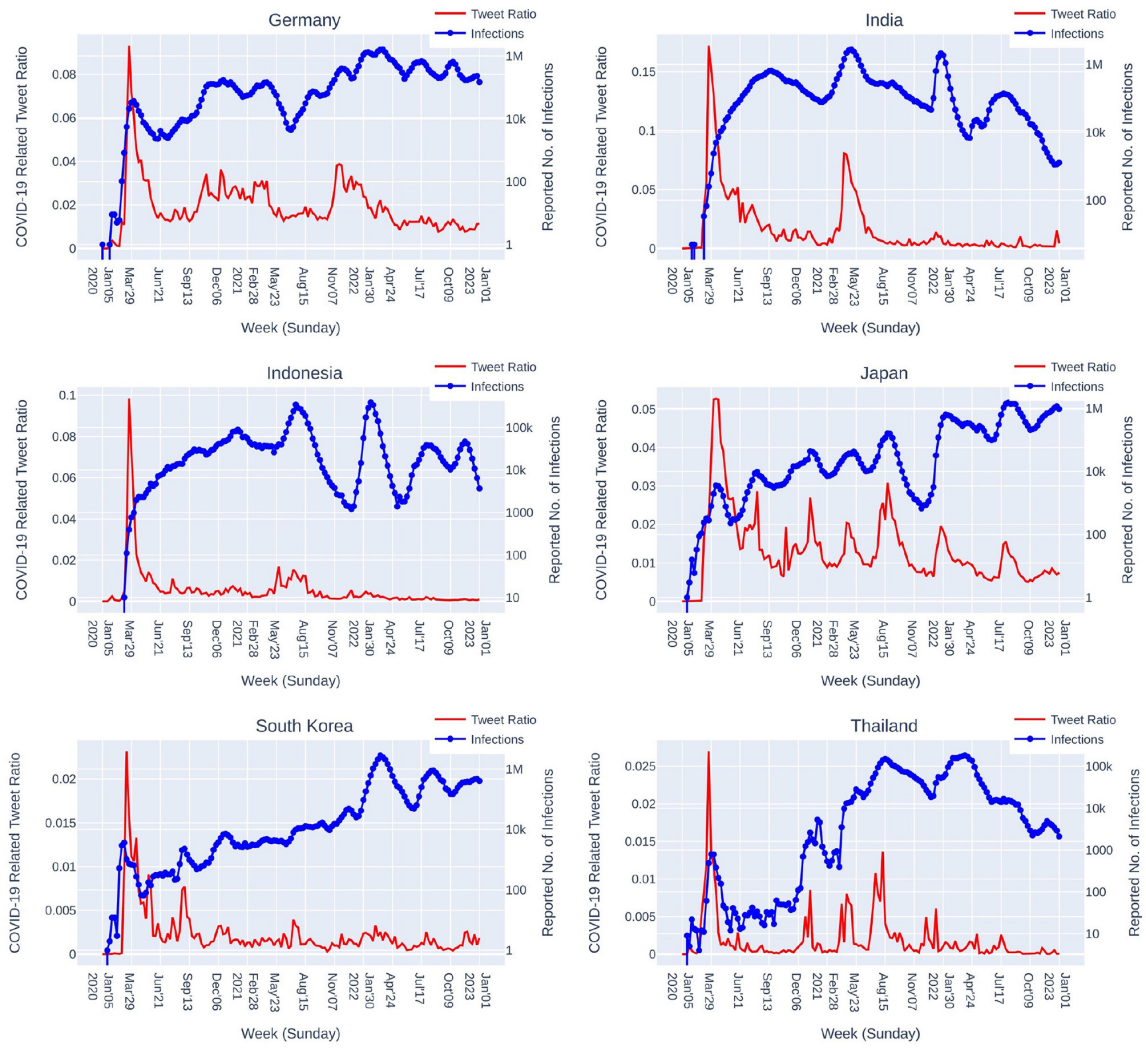
COVID-19 related social media (Twitter) reaction depicted by the ratio of weekly total tweet counts of “COVID-19 related tweets” vs. “all tweets” since January 2020. The reported number of infections is plotted in weekly sum.

As shown in Figure 8, the long term tendencies of social media reaction on Twitter among the 6 countries are quite similar. The reaction dramatically surged in the beginning of the COVID-19 pandemic and quickly made a steep drop afterward. In 2021, each country faced new waves at different timing and so the social media reaction raised again. Noticeably as also shown in Table 4, in 2022, relatively much lower level of social media reaction was observed in all countries. In general, social media reaction toward the COVID-19 pandemic dramatically surged in the beginning and gradually faded out overtime.

**Table 4:** COVID-19 related social media (Twitter) reaction depicted by the ratio of annually total tweet counts of “COVID-19 related tweets” vs. “all tweets.”

	**Germany**	**India**	**Indonesia**	**Japan**	**South Korea**	**Thailand**
2020	2.32%	3.23%	1.01%	1.70%	0.37%	0.23%
2021	2.20%	1.69%	0.56%	1.37%	0.14%	0.27%
2022	1.38%	0.38%	0.15%	0.91%	0.14%	0.07%

Though having quite similar tendencies, the magnitudes of the social media reaction surges are somewhat different among the 6 countries. Comparing to the beginning of COVID-19, Japan, Germany, and Thailand exhibit several times relatively higher magnitudes of the social reaction surges comparing to India (one time high-magnitude surge), and the two countries, Indonesia and South Korea, with relatively low magnitudes of the social media reaction.

### 4.2. COVID-19 case prediction

The results of COVID-19 case prediction for Germany, India, Indonesia, South Korea and Thailand as shown in Table 5 demonstrate that using emoji features yields better prediction performance in most of the cases and worse performance in only 3 cases out of 192 cases. Although with differences among the 6 countries appearing in the percentage of emoji usage in COVID-19 related tweets (Germany: 4.08%, India: 8.27%, Indonesia: 8.02%, Japan: 8.66%, South Korea: 2.84%, and Thailand: 4.60%), and the ratio of COVID-19 related social media reaction over general social media reaction (Figure 8 and Table 4), COVID-19 related tweets containing emoji can provide informative features contributing to the improvement of our COVID-19 case prediction system.

**Table 5:** Evaluation results of our system for 6 countries with the emoji features with Twitter's “context annotation”.

		**Window = 7**		**Window = 14**		**Window = 21**		**Window = 28**	
	**Period**	**B**	**E**	**B**	**E**	**B**	**E**	**B**	**E**
**Germany**	1	8.8(6.1)	**6.8**(5.7)	17.8(12.3)	**13.9**(10.1)	24.4(14.0)	**20.9**(14.6)	29.9(17.0)	**26.4**(18.5)
	2	12.8(9.5)	**10.0**(5.6)	26.0(16.1)	**20.5**(10.7)	35.4(20.8)	**29.6**(14.9)	42.4(22.5)	**36.4**(18.2)
	3	17.9(9.5)	**14.1**(8.7)	40.5(23.8)	**30.0**(19.8)	71.7(48.0)	**52.7**(36.0)	120.3(93.6)	**78.5**(51.8)
	4	8.8(6.6)	**8.4**(6.0)	18.4(10.7)	**16.9**(10.2)	27.7(12.9)	**25.0**(12.8)	34.6(14.9)	**32.5**(14.6)
	5	8.6(5.8)	**8.2**(6.3)	18.6(11.5)	**16.4**(10.0)	26.4(13.1)	**25.0**(11.5)	32.6(12.3)	**32.5**(11.4)
	6	8.6(6.7)	**7.8**(6.1)	15.4(11.0)	**14.8**(10.5)	21.4(14.6)	**20.9**(14.2)	27.0(17.2)	**26.7**(17.2)
	7	17.7(8.5)	**16.3**(8.7)	37.6(14.9)	**32.2**(13.1)	61.3(28.0)	**50.8**(20.7)	81.9(45.9)	**71.1**(38.3)
	8	9.2(6.9)	**7.9**(5.9)	18.1(13.1)	**16.1**(11.2)	29.6(18.2)	**25.6**(16.4)	40.9(23.8)	**36.8**(20.7)
**India**	1	8.4(6.0)	**7.0**(5.3)	13.7(7.0)	**12.5**(6.2)	18.8(9.3)	**18.4**(8.0)	26.7(12.9)	**25.2**(10.6)
	2	6.5(4.4)	**6.4**(4.9)	**13.4**(8.4)	13.5(8.5)	19.8(11.3)	**19.0**(11.2)	26.7(13.7)	**25.3**(13.8)
	3	7.3(5.2)	**5.9**(4.2)	19.4(12.2)	**15.7**(11.4)	42.8(28.5)	**30.7**(24.0)	81.7(53.7)	**58.7**(41.9)
	4	8.1(7.4)	**6.9**(6.3)	13.3(14.9)	**12.3**(12.6)	17.7(18.8)	**16.5**(16.9)	21.9(20.5)	**19.6**(19.8)
	5	8.8(9.0)	**8.0**(9.0)	22.6(16.9)	**19.0**(15.7)	31.8(19.1)	**27.4**(18.4)	37.6(19.7)	**32.8**(19.7)
	6	35.7(25.6)	**19.9**(14.8)	171.9(136.9)	**66.9**(45.3)	456.0(381.7)	**177.9**(120.4)	798.1(644.2)	**377.2**(284.6)
	7	11.6(8.8)	**9.9**(7.9)	24.0(13.0)	**21.1**(11.7)	32.8(15.8)	**30.1**(14.3)	36.2(15.5)	**34.5**(14.8)
	8	7.3(4.6)	**6.1**(4.4)	17.3(11.7)	**15.1**(11.4)	30.2(21.6)	**27.1**(20.7)	51.4(32.6)	**44.4**(32.4)
**Indonesia**	1	7.7(5.2)	**7.0**(5.0)	**12.1**(7.2)	12.4(8.4)	16.6(7.2)	**16.2**(9.5)	19.8(7.0)	**19.3**(9.5)
	2	8.7(6.4)	**8.5**(6.0)	15.8(9.1)	**14.5**(9.3)	23.1(10.8)	**20.4**(11.2)	30.9(14.0)	**27.3**(14.5)
	3	12.2(9.4)	**9.4**(7.0)	17.6(12.1)	**15.1**(11.1)	21.2(15.3)	**19.0**(14.1)	24.9(18.4)	**22.8**(16.8)
	4	12.8(7.7)	**11.1**(6.7)	35.4(11.8)	**24.8**(11.9)	74.1(35.8)	**47.6**(25.6)	159.0(86.7)	**93.6**(58.2)
	5	10.4(7.0)	**9.8**(6.8)	19.3(10.3)	**17.9**(10.9)	32.9(13.5)	**26.1**(12.0)	58.5(28.7)	**36.7**(11.6)
	6	23.7(12.3)	**20.6**(12.0)	71.7(45.7)	**46.9**(25.9)	190.6(174.7)	**112.3**(79.9)	405.3(432.7)	**281.9**(265.7)
	7	18.8(13.4)	**15.7**(12.6)	52.0(42.0)	**30.0**(17.6)	135.2(155.4)	**55.1**(46.3)	300.8(430.8)	**96.7**(116.9)
	8	6.8(4.6)	**6.3**(4.0)	15.6(7.9)	**14.2**(7.6)	25.4(13.1)	**23.1**(12.1)	40.8(20.0)	**34.9**(17.8)
**Japan**	1	7.8(5.1)	**6.7**(4.7)	13.3(6.4)	**11.5**(6.6)	20.5(9.9)	**18.0**(8.3)	26.5(11.2)	**24.3**(9.3)
	2	16.5(11.7)	**14.1**(10.2)	38.3(31.7)	**34.1**(28.5)	62.9(52.9)	**59.4**(51.5)	87.9(74.8)	**83.8**(72.7)
	3	13.3(12.1)	**9.2**(7.8)	23.6(22.8)	**17.6**(15.4)	32.8(32.5)	**26.5**(24.3)	44.5(41.6)	**37.3**(34.9)
	4	17.3(11.5)	**14.3**(9.6)	45.3(36.8)	**38.3**(29.3)	93.7(90.9)	**75.0**(69.7)	204.3(225.4)	**144.9**(142.1)
	5	14.2(7.3)	**11.9**(6.8)	37.6(22.1)	**26.0**(13.3)	76.9(67.4)	**40.7**(23.3)	184.4(194.6)	**78.0**(74.2)
	6	11.9(12.4)	**11.0**(12.2)	21.3(15.5)	**17.8**(16.3)	33.2(18.0)	**26.1**(18.3)	45.0(23.6)	**36.7**(22.6)
	7	17.0(12.5)	**11.8**(10.4)	29.9(22.7)	**22.6**(17.3)	39.2(26.3)	**30.9**(21.4)	47.0(26.3)	**37.8**(23.5)
	8	19.7(18.5)	**11.4**(7.9)	34.6(23.7)	**22.7**(14.2)	50.6(32.3)	**37.2**(21.0)	76.3(51.2)	**58.2**(33.1)
**South Korea**	1	9.3(6.3)	**8.8**(5.5)	17.6(9.8)	**17.0**(9.1)	26.4(13.3)	**25.6**(12.3)	34.2(17.7)	**33.8**(18.7)
	2	8.6(5.9)	**7.8**(5.6)	15.8(15.8)	**14.9**(13.4)	22.7(24.1)	**21.6**(21.4)	28.9(28.8)	**26.3**(26.5)
	3	8.8(5.8)	**6.9**(4.6)	12.8(8.4)	**10.7**(7.4)	14.8(9.8)	**13.3**(9.7)	16.8(11.2)	**15.6**(11.3)
	4	7.8(7.6)	**6.8**(6.5)	14.3(13.9)	**11.0**(10.5)	16.8(15.4)	**13.2**(11.7)	18.5(15.6)	**14.8**(12.6)
	5	14.8(10.7)	**11.3**(7.7)	25.8(11.5)	**22.9**(12.6)	31.8(11.9)	**31.0**(15.2)	**34.0**(12.3)	35.4(17.1)
	6	11.5(7.5)	**11.1**(7.5)	24.5(13.0)	**22.9**(13.5)	37.7(14.6)	**35.3**(17.6)	49.6(20.2)	**48.9**(26.3)
	7	15.8(9.9)	**11.6**(8.8)	43.5(26.2)	**29.0**(18.8)	90.7(58.8)	**61.2**(39.8)	170.2(101.0)	**112.4**(73.2)
	8	14.0(9.0)	**12.7**(9.1)	27.1(15.5)	**25.2**(13.8)	47.7(29.0)	**40.8**(23.7)	69.7(43.8)	**59.7**(37.7)
**Thailand**	1	30.1(27.2)	**26.9**(29.0)	35.1(24.7)	**33.6**(28.8)	40.2(23.3)	**38.9**(27.2)	45.3(25.0)	**43.5**(27.3)
	2	89.3(120.5)	**67.1**(83.3)	141.7(191.6)	**124.4**(167.0)	175.2(221.0)	**151.7**(191.6)	177.0(208.3)	**158.8**(186.8)
	3	23.1(13.9)	**18.7**(13.2)	30.6(17.2)	**26.3**(16.9)	35.6(18.2)	**31.5**(17.9)	38.7(18.9)	**35.4**(18.7)
	4	7.6(5.8)	**5.9**(4.5)	15.3(8.9)	**11.9**(7.1)	23.3(10.1)	**18.1**(7.8)	30.6(11.4)	**24.4**(8.8)
	5	4.3(3.2)	**4.2**(3.3)	10.2(7.4)	**9.8**(7.9)	18.5(11.0)	**16.2**(11.6)	27.0(12.9)	**23.0**(14.6)
	6	9.4(8.8)	**7.9**(8.2)	15.2(13.5)	**14.4**(12.8)	19.1(15.2)	**18.1**(14.3)	23.3(16.0)	**21.8**(15.2)
	7	10.3(6.3)	**9.6**(6.4)	25.1(16.1)	**22.1**(14.2)	41.4(28.1)	**35.6**(22.1)	61.9(42.8)	**49.9**(34.2)
	8	12.0(11.9)	**10.2**(10.5)	27.8(28.8)	**21.3**(22.6)	34.2(34.9)	**28.3**(27.6)	45.2(47.2)	**40.9**(41.1)

The values are mean and standard deviation (in parentheses) of MRAE of the prediction windows starting at the dates in each 3-month period of 1) Oct-Dec 2020, 2) Jan-Mar 2021, 3) Apr-Jun 2021, 4) Jul-Sep 2021, 5) Oct-Dec 2021, 6) Jan-Mar 2022, 7) Apr-Jun 2022, and 8) Jul-Sep 2022. Notations: ***B*** - bare system not using emoji features, ***E*** - system using emoji features. In most of the cases, except 3, the system using emoji features (***B***) perform better. The error values are shown in percentage with % omitted. The bold values indicate better performance.

As seen in Table 5, we can observe several noticeable abnormal errors larger than 100%, for instance, 〈 India, Period 6 (Jan-Mar 2022), Window = 28 〉 with 798.1% for the bare system not using emoji features ***B*** and 377.2% for the system using emoji features ***E***. Based on Equation 17, the error value of 798.1% indicates that the predicted number of cases is about 9 times of the observed number of cases. In analyzing this situation, we see that, in the mentioned period, India was in an unprecedented epidemic wave when the raising and dropping of the number of cases were dramatic in a relatively shorter period of time comparing to the previous waves (Figure 8). While the higher speed of raising number of cases could be attributed to a newer variant, the dropping speed was also higher. It took 21 days since 2022-01-26 (the peak of the wave in Period 6) for the (smoothed) number of cases to drop about ≈7 times, while for the same dropping ratio of ≈7 times, it took 45 days since 2021-05-09 (the previous peak), and 78 days since 2022-07-23 (the next peak). Even though our system equipped with emoji features managed to cut the error to 377.2%, this is still a considerable error. Similar situations are also observed in other countries. In these situations, the prediction error gets magnified at a greater rate as we try to predict further into the future with a larger window size. The system had a hard time to adapt to a dramatic change of situation where past data do not contain adequate information.

## 5. Discussion

The results of our study on utilizing social media data for constructing a COVID-19 case prediction system suggests that using social media can be helpful for epidemic forecasting. In terms of prediction accuracy, our experimental results show that using both tweet count and anomalous score improved the MRAE of COVID-19 case prediction. In terms of practicality, the utilization of emoji as a means of shallow emotion analysis can be easily applied to multilingual social media platforms worldwide. In addition to the use of tweet count and anomalous score, a future direction is to investigate higher dimension representation of emoji, for instance, by utilizing EmojiNet (33), a dictionary of emoji senses, to represent emoji in a high-dimensional vector space of semantics.

As shown by the change in social media reaction over the long course of the COVID-19 epidemic in Japan, behavioral changes may differ remarkably wave-to-wave, which challenges our system's ability to adapt and perform well. The proportion of social media attention to COVID-19 vs. general topics on Twitter was only 32 and 22% in the 6th and 7th waves relative to that in the 1st wave whereas it was 56–62% in the 2nd to 5th waves. One of the major factors in these differences was governmental policies: the Japan government did not declare a state of emergency during three waves: the 2nd, 6th, and 7th. In the 2nd wave immediately after the 1st wave, most people may have realized that the COVID-19 epidemic was not over. Therefore, while COVID-19 was still somewhat mysterious, there was not much surprise when the 2nd wave hit. As a result, although there was social media reaction, it dropped more than half (Figure 4). When the 3rd, 4th, and 5th waves hit, the social media reaction proportion was around 30s%, similar to that for the 2nd. Our system performed well for the 3rd and 4th waves, but not for the 5th one. This is attributed to much higher morbidity in the 5th wave, to which our system could not adapt adequately. Even after learning from the change in the 5th wave, our system performed worse in the 6th wave, which was characterized by an even higher level of morbidity and a marked difference in government policy: a state of emergency was not declared. Our system was able to learn from that change and performed better for the 7th wave, which again came with another higher level of morbidity and no declaration of a state of emergency.

Systems for epidemic forecasting that are based on only machine learning and historical data may be limited and suffer from unstable system performance when the epidemic lasts long enough to be characterized by several waves and different governmental policies, public perceptions, and attitudes. Our experimental results suggest that maintaining performance in later waves of an epidemic is a challenge. Machine-learning-based systems may need more data in order to adequately learn about social changes and thereby maintain performance. Future work should consider situations that are difficult to characterize from past data for constructing prediction system based on machine learning.

If social media attention on an epidemic starts to fade, it may be helpful to look at social media signals other than those directly related to the epidemic as an aid to forecasting systems. Although social media attention in Japan on COVID-19 has declined over the course of the epidemic, social media activity related to general topics grew by 280% in the 7th wave compared with that in the 1st wave (Figure 4), a period of slightly less than 3 years. Although looking at social media signals other than those directly related to the epidemic seems to be a promising approach in term of data volume, it is challenging in term of data collection and processing. This is because data containing social media reactions to all kinds of topics and problems is unrestricted and difficult to control. Even though a restricted set of pandemic-related social media data is more focused and can directly help obtain valuable knowledge about the public issues during a pandemic, for instance, social health problems including stress, fear, and anxiety (13–15), a decrease in such social media data makes it more challenging to analyze those problems. Hence, expanding the scope of social media data analysis beyond pandemic-related topics while keeping high-quality analysis is necessary and challenging in dealing with a long-lasting pandemic.

Our results show the potential of using emoji as a proxy for public social media emotion analysis in predicting the progression of a pandemic, which suggests the potential of monitoring public emotion for the task. On the one hand, for per-patient monitoring, medical big data and wearable Internet of Medical Things (34–36) provide the ability to monitor the physical conditions of patients directly and aid them individually and privately in real time. However, such data is valuable and may not be shared across regions without strict regulations. On the other hand, public social media data analysis can help with monitoring in real time the public in terms of critical aspects, for instance, emotions, which can support cross-region analyses. Emoji can be easily monitored across all public social media platforms in all languages at low cost and thereby support shallow emotion analysis. Though multilingual language models can also be adapted for emotion analysis, access to public social media platforms is limited by the access rate, making mass full-text access difficult, which poses a challenge for mass and deep emotion analysis.

The different interpretations of the meaning of emoji make it difficult to use emoji to correctly interpret the true underlying emotion of social media users in general and toward COVID-19 in particular. On the bright side, studies showed that large similarity exists in interpreting emoji meanings in age-based and nation-based analyses. Gallud et al. (37), though a questionnaire, found that older people did not have a lesser understanding of emoji than young people, though, there were also results indicating the varying understandings. In the questionnaire, which asked for the meaning of emoji referred in Emojipedia ^12^, some emoji got answers with high accuracy (>80%), some got answers with low accuracy (<30%). The “fear” emoji (17.1% accuracy) was also answered as “surprise.” Also in the work of Kutsuzawa et al. (38), they found that, for both young and middle-aged groups, in general, emoji were similarly clustered in Arousal-Valence space, but some emoji were interpreted differently among different age groups. Schouteten et al. (39) found that emoji meanings (pleasure-arousal-dominance dimensions) are largely similar in 5 countries (Germany, Singapore, Malaysia, UK, and New Zealand). Still, misunderstandings of emoji were also observed (40), for instance, “praying hands” misunderstood as “a high five,” “irritation, anger, and contempt” misunderstood as “pride face,” and “confused” misunderstood as “frustrated and sad face.” Despite the results of large similarity in interpreting emoji meanings, deeper interpretation of the true underlying emotion is challenging. In a systematic review of research on emoji, Bai et al. (41) stated that, “at present, it is difficult to accurately measure participants' true reactions through self-reporting. Categorizing emotions by amassing a corpus using big data is unable to depict users' complex emotions such as are expressed by emoji at a more detailed level, for example emotions such as shame, anger and so on.” They suggested that “observing whether users' actual facial expressions differ from their selected emoji emotionally in communication can help researchers understand users' psychological mechanism in communication”. In the light of those studies, while we have found evidence for the effectiveness of using emoji features in improving COVID-19 case prediction system, much deeper analysis of social media users' true underlying emotions which significantly affect their behaviors is a difficult challenge.

This work has these limitations:

- Even though Twitter is a super popular and influential social media platform, not a majority of the population of each of the mentioned countries have a Twitter account or actively use the platform. Therefore, the collected data are not coming from the whole population. While the data could be seen as influential social media signal as also shown in our results, it should be treated with caution when representing the general population's reaction.
- Only COVID-19 related tweets are considered. As shown above in the data from the 6 countries, COVID-19 related tweets only constitute a small potion of social media. Arik et al. (32) showed that it is necessary to study factors, e.g., per capita income, hospital patient experience rating, and air quality measures, which affects the epidemic progression. It is then intuitive to expand the study to social media reaction over those topics, for example, economics, and climate, together with COVID-19.
- Emoji is used as the sole proxy to capture social media emotion reaction. Even though our study showed positive contribution of emoji features, emoji usage is still relatively small with less than 10% given the data from the 6 countries. For larger coverage of social media emotion analysis, full-text-based analysis could be considered for platforms where full-text access at large scale is feasible, especially when considering other topics together with COVID-19. Even though Twitter API provides Sample Stream API which can be used for collecting 1 or 10% random tweets, biases in the sampling method were reported (42, 43) and exploitable (44).
- Location-based social media data collection is difficult. It will even be more so in the future when privacy issues will be even more recognized and respected. This work uses language to collect country-based data, which is applicable to only some countries. For future utilization of social media data, location sharing policy should be more fine-grained managed, for example, letting users select a lower precision level of location to share only the city or state they are in.

## 6. Conclusion

We have investigated the use of emoji as a proxy for estimating social media reaction in terms of emotion trends on Twitter with the aim of constructing a system for predicting the daily number of COVID-19 cases in Japan. Our experimental results showed that using emoji features improves system performance. These experimental results together with our analysis of Twitter data suggest that prediction of the later waves of an epidemic could be more challenging. The difficulty may be related to changes in both the epidemic characteristics (variants and their properties) and social media reaction. Future work should consider situations that are difficult to characterize from historical data for constructing prediction system based on machine learning.

## Data availability statement

The data analyzed in this study is subject to the following licenses/restrictions: The COVID-19 infection reporting data for Japan were publicly provided by Japanese Ministry of Health, Labor and Welfare (https://covid19.mhlw.go.jp/en/). The COVID-19 infection reporting data for Germany, India, Indonesia, South Korea, and Thailand were publicly provided by the World Health Organization (https://covid19.who.int/data). The Twitter data analyzed in this study were obtained from Twitter and used in accordance with the licenses and restrictions of Twitter's Developer Agreement and Policy. Requests to access these datasets should be directed to https://twitter.com/.

## Author contributions

VT and TM contributed to the conception and design of the study and to the data collection. VT implemented the system, performed data curation, conducted the experiments, and wrote the first draft of the manuscript. TM validated the progress and results of the study *via* daily discussion with VT. All authors contributed to manuscript revision and read and approved the submitted version.

## Footnotes

^1^ https://www.statista.com/statistics/242606/number-of-active-twitter-users-in-selected-countries/

^2^ https://www.bigbeatinc.com/blog/japan_social_media_2022

^3^ https://covid19.mhlw.go.jp/en/

^4^ https://covid19.who.int/data

^5^Twitter API context annotation for COVID-19: { domain: { id: 123 }, entity: { id: 1220701888179359745, name: COVID-19 } }.

^6^ https://developer.twitter.com/en/docs/twitter-api/annotations/faq

^7^ https://help.twitter.com/en/safety-and-security/tweet-location-settings

^8^“lang” codes: de (German, Germany), hi (Hindi, India), id (Indonesian, Indonesia), ja (Japanese, Japan), ko (Korean, South Korea), and th (Thai, Thailand).

^9^ https://www.statista.com/statistics/242606/number-of-active-twitter-users-in-selected-countries/

^10^ https://console.cloud.google.com/marketplace/product/bigquery-public-datasets/covid19-public-forecasts

^11^ https://developer.twitter.com/en/docs/twitter-api/tweet-caps

^12^ http://emojipedia.org

## References

[1] Neely S Eldredge C Sanders R. Health information seeking behaviors on social media during the COVID-19 pandemic among American social networking site users: survey study. J Med Internet Res. (2021) 23:e29802. 10.2196/29802 34043526 PMC8202660

[2] Dadaczynski K Okan O Messer M Leung AYM Rosário R Darlington E. Digital health literacy and web-based information-seeking behaviors of university students in Germany during the COVID-19 pandemic: cross-sectional survey study. J Med Internet Res. (2021) 23:e24097. 10.2196/24097 33395396 PMC7813561

[3] Settanni M Marengo D. Sharing feelings online: studying emotional well-being *via* automated text analysis of Facebook posts. Front Psychol. (2015) 6:1045. 10.3389/fpsyg.2015.01045 26257692 PMC4512028

[4] Park M Cha C Cha M. Depressive moods of users portrayed in Twitter. In: Proceedings of the ACM SIGKDD Workshop on Healthcare Informatics (HI-KDD). Beijing (2012). p. 1–8.

[5] Wald R Khoshgoftaar TM Napolitano A Sumner C. Using Twitter content to predict psychopathy. In: 2012 11th International Conference on Machine Learning and Applications. Vol. 2. Boca Raton, FL: IEEE (2012). p. 394–401. 10.1109/ICMLA.2012.228

[6] Goldberg LR. An alternative “description of personality”: the big-five factor structure. J Pers Soc Psychol. (1990) 59:1216. 10.1037/0022-3514.59.6.1216 2283588

[7] Jones DN Paulhus DL. Introducing the short dark triad (SD3) a brief measure of dark personality traits. Assessment. (2014) 21:28–41. 10.1177/1073191113514105 24322012

[8] Wheaton MG Prikhidko A Messner GR. Is fear of COVID-19 contagious? The effects of emotion contagion and social media use on anxiety in response to the coronavirus pandemic. Front Psychol. (2021) 11:3594. 10.3389/fpsyg.2020.567379 33469434 PMC7813994

[9] Arora A Chakraborty P Bhatia M Mittal P. Role of emotion in excessive use of Twitter during COVID-19 imposed lockdown in India. J Technol Behav Sci. (2021) 6:370–77. 10.1007/s41347-020-00174-3 33102690 PMC7572156

[10] Kaur S Kaul P Zadeh PM. Monitoring the dynamics of emotions during COVID-19 using Twitter data. Procedia Comput Sci. (2020) 177:423–30. 10.1016/j.procs.2020.10.056

[11] Toriumi F Sakaki T Yoshida M. Social emotions under the spread of COVID-19 using social media. Trans Jpn Soc Artif Intell. (2020) 35:F-K45_1-7. 10.1527/tjsai.F-K45

[12] Dyer J Kolic B. Public risk perception and emotion on Twitter during the Covid-19 pandemic. Appl Netw Sci. (2020) 5:99. 10.1007/s41109-020-00334-7 33344760 PMC7739810

[13] Lăzăroiu G Horak J Valaskova K. Scaring ourselves to death in the time of COVID-19: pandemic awareness, virus anxiety, and contagious fear. Linguist Philos Invest. (2020) 19:114–20. 10.22381/LPI1920208

[14] Rommer D Majerova J Machova V. Repeated COVID-19 pandemic-related media consumption: minimizing sharing of nonsensical misinformation through health literacy and critical thinking. Linguist Philos Invest. (2020) 19:107–13. 10.22381/LPI1920207

[15] Lăzăroiu G Adams C. Viral panic and contagious fear in scary times: The proliferation of COVID-19 misinformation and fake news. Anal Metaphys. (2020) 19:80–6. 10.22381/AM1920209

[16] Ljungholm DP Olah ML. Regulating fake news content during COVID-19 pandemic: evidence-based reality, trustworthy sources, and responsible media reporting. Rev Contemporary Philos. (2020) 19:43–9. 10.22381/RCP1920203

[17] Suntwal S Brown S Brandimarte L. Pictographs, Ideograms, and Emojis (PIE): A Framework for Empirical Research Using Non-verbal Cues. In: Proceedings of the 54th Hawaii International Conference on System Sciences. Kauai, HI (2021). p. 6400. 10.24251/HICSS.2021.771

[18] Elder AM. What words can't say: emoji and other non-verbal elements of technologically-mediated communication. J Inf Commun Ethics Soc. (2018) 16:2–15. 10.1108/JICES-08-2017-0050

[19] Cheng L. Do i mean what i say and say what i mean? A cross cultural approach to the use of emoticons & emojis in CMC messages. Fonseca J Commun. (2017) 15:199–217. 10.14201/fjc201715199217

[20] Lo SK. The nonverbal communication functions of emoticons in computer-mediated communication. Cyberpsychol Behav. (2008) 11:595–7. 10.1089/cpb.2007.0132 18817486

[21] Rosenthal S Farra N Nakov P. SemEval-2017 task 4: Sentiment analysis in Twitter. In: Proceedings of the 11th International Workshop on Semantic Evaluation (SemEval-2017). Vancouver, BC (2017). p. 502–18. 10.18653/v1/S17-2088

[22] Dini L Bittar A. Emotion analysis on twitter: the hidden challenge. In: Proceedings of the Tenth International Conference on Language Resources and Evaluation (LREC'16). Portorož (2016). p. 3953–8.

[23] Hochreiter S Schmidhuber J. Long short-term memory. Neural Comput. (1997) 9:1735–80. 10.1162/neco.1997.9.8.1735 9377276

[24] Yousefinaghani S Dara R Mubareka S Sharif S. Prediction of COVID-19 waves using social media and google search: a case study of the US and Canada. Front Public Health. (2021) 9:359. 10.3389/fpubh.2021.656635 33937179 PMC8085269

[25] Azzaoui AE Singh SK Park JH. SNS big data analysis framework for COVID-19 outbreak prediction in smart healthy city. Sustain Cities Soc. (2021) 71:102993. 10.1016/j.scs.2021.102993 33996386 PMC8103782

[26] Chew AWZ Pan Y Wang Y Zhang L. Hybrid deep learning of social media big data for predicting the evolution of COVID-19 transmission. Knowl Based Syst. (2021) 233:107417. 10.1016/j.knosys.2021.107417 34690447 PMC8522122

[27] Tran V Matsui T. Tweet analysis for enhancement of COVID-19 epidemic simulation: a case study in Japan. Front Public Health. (2022) 10:806813. 10.3389/fpubh.2022.806813 35433607 PMC9008370

[28] Bindu PV Thilagam PS. Mining social networks for anomalies: methods and challenges. J Netw Comput Appl. (2016) 68:213–29. 10.1016/j.jnca.2016.02.021

[29] Rossi RA Gallagher B Neville J Henderson K. Modeling dynamic behavior in large evolving graphs. In: Proceedings of the Sixth ACM International Conference on Web Search and Data Mining. WSDM '13. New York, NY: Association for Computing Machinery (2013). p. 667–76. 10.1145/2433396.2433479

[30] Henderson K Gallagher B Li L Akoglu L Eliassi-Rad T Tong H. It's who you know: graph mining using recursive structural features. In: Proceedings of the 17th ACM SIGKDD International Conference on Knowledge Discovery and Data Mining. San Diego, CA (2011). p. 663–71. 10.1145/2020408.2020512

[31] Yu Y Si X Hu C Zhang J. A review of recurrent neural networks: LSTM cells and network architectures. Neural Comput. (2019) 31:1235–70. 10.1162/neco_a_01199 31113301

[32] Arik S Li CL Yoon J Sinha R Epshteyn A Le L. Interpretable sequence learning for COVID-19 forecasting. Adv Neural Inf Process Syst. (2020) 33:18807–18. 36266338

[33] Wijeratne S Balasuriya L Sheth A Doran D. Emojinet: an open service and api for emoji sense discovery. In: Eleventh International AAAI Conference on Web and Social Media. Montreal, QC (2017). 10.1609/icwsm.v11i1.14857

[34] Hurley D Popescu GH. Medical big data and wearable internet of things healthcare systems in remotely monitoring and caring for confirmed or suspected COVID-19 patients. Am J Med Res. (2021) 8:78–90. 10.22381/ajmr8220216

[35] Welch H Michalikova KF. Artificial intelligence-powered diagnostic tools, networked medical devices, and cyber-physical healthcare systems in assessing and treating patients with COVID-19 symptoms. Am J Med Res. (2021) 8:91–104. 10.22381/ajmr8220217

[36] Turner D Pera A. Wearable internet of medical things sensor devices, big healthcare data, and artificial intelligence-based diagnostic algorithms in real-time COVID-19 detection and monitoring systems. Am J Med Res. (2021) 8:132–45. 10.22381/ajmr82202110

[37] Gallud JA Fardoun HM Andres F Safa N. A study on how older people use emojis. In: Proceedings of the XIX International Conference on Human Computer Interaction. Palma (2018). p. 1–4. 10.1145/3233824.3233861

[38] Kutsuzawa G Umemura H Eto K Kobayashi Y. Age differences in the interpretation of facial emojis: classification on the arousal-valence space. Front Psychol. (2022) 13:915550. 10.3389/fpsyg.2022.915550 35910971 PMC9333063

[39] Schouteten JJ Llobell F Chheang SL Jin D Jaeger SR. Emoji meanings (pleasure-arousal-dominance dimensions) in consumer research: between-country and interpersonal differences. J Food Sci. (2022) 2022:16374. 10.1111/1750-3841.16374 36413025

[40] Park J Baek YM Cha M. Cross-cultural comparison of nonverbal cues in emoticons on Twitter: evidence from big data analysis. J Commun. (2014) 64:333–54. 10.1111/jcom.12086

[41] Bai Q Dan Q Mu Z Yang M. A systematic review of emoji: current research and future perspectives. Front Psychol. (2019) 10:2221. 10.3389/fpsyg.2019.02221 31681068 PMC6803511

[42] Joseph K Landwehr PM Carley KM. Two 1% s don't make a whole: comparing simultaneous samples from Twitter's streaming API. In: International Conference on Social Computing, Behavioral-Cultural Modeling, and Prediction. Springer (2014). p. 75–83. 10.1007/978-3-319-05579-4_10

[43] Morstatter F Pfeffer J Liu H. When is it biased? Assessing the representativeness of twitter's streaming API. In: *Proceedings of the 23rd International Conference on World Wide Web*. Seoul (2014). p. 555–6. 10.1145/2567948.2576952

[44] Pfeffer J Mayer K Morstatter F. Tampering with Twitter's sample API. EPJ Data Sci. (2018) 7:50. 10.1140/epjds/s13688-018-0178-0

