# Supplementary material for: COVID-19 case prediction using emotion trends via Twitter emoji analysis: A case study in Japan
Source: Front Public Health. 2023 Mar 14;11:1079315. doi: 10.3389/fpubh.2023.1079315 (PMC10045477; doi:10.3389/fpubh.2023.1079315)
Supplement: Supplementary file 1 [file Data_Sheet_1.pdf]

Supplementary Material

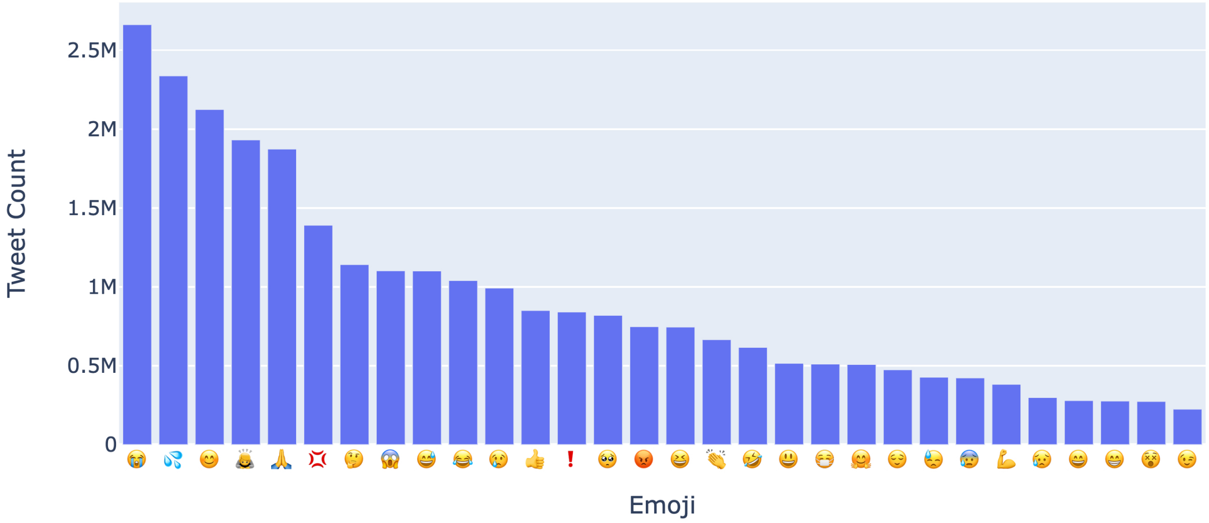

Figure S1. COVID-19 related tweet counts using our pre-defined keywords for the 30 emoji used in this study.

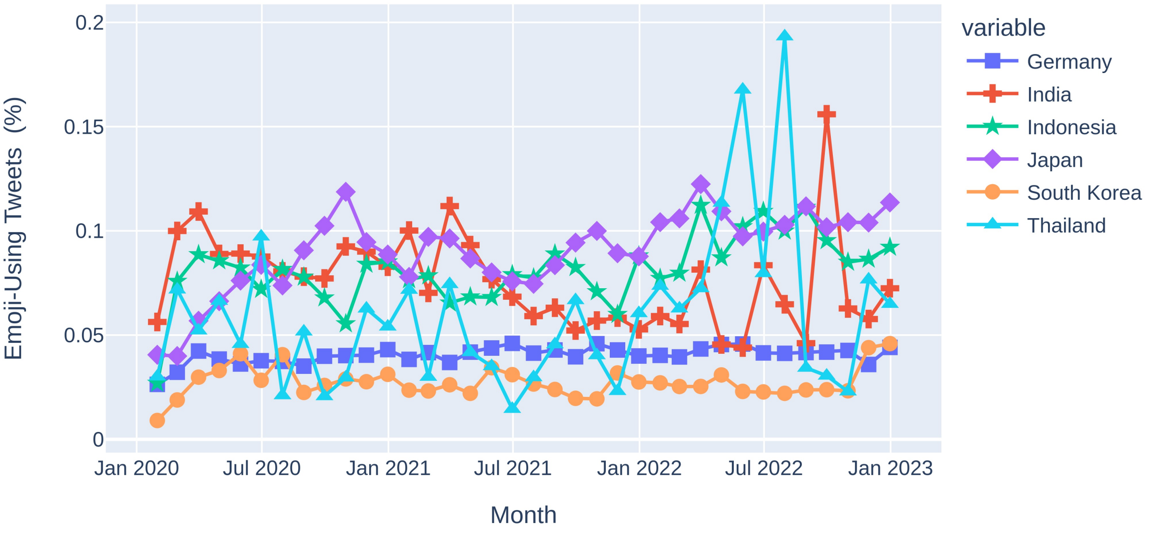

Figure S2. Monthly ratio of emoji-using tweets in COVID-19 related tweets categorized by Twitter API's "context annotation" function for 6 countries.

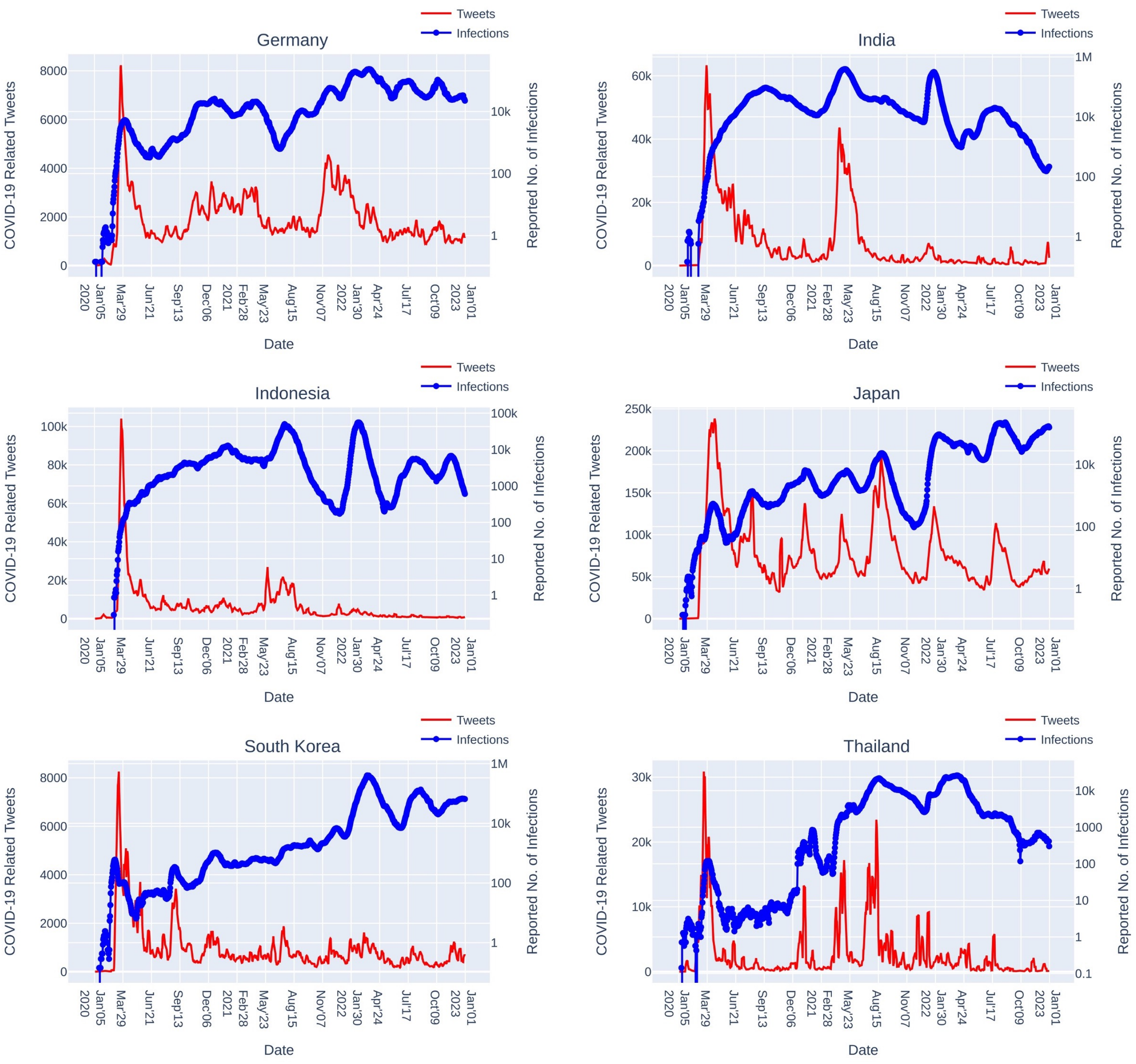

Figure S3. Tweet counts of COVID-19 related tweets categorized by Twitter API's "context annotation" function for 6 countries. The values are smoothed with 7-day moving average.

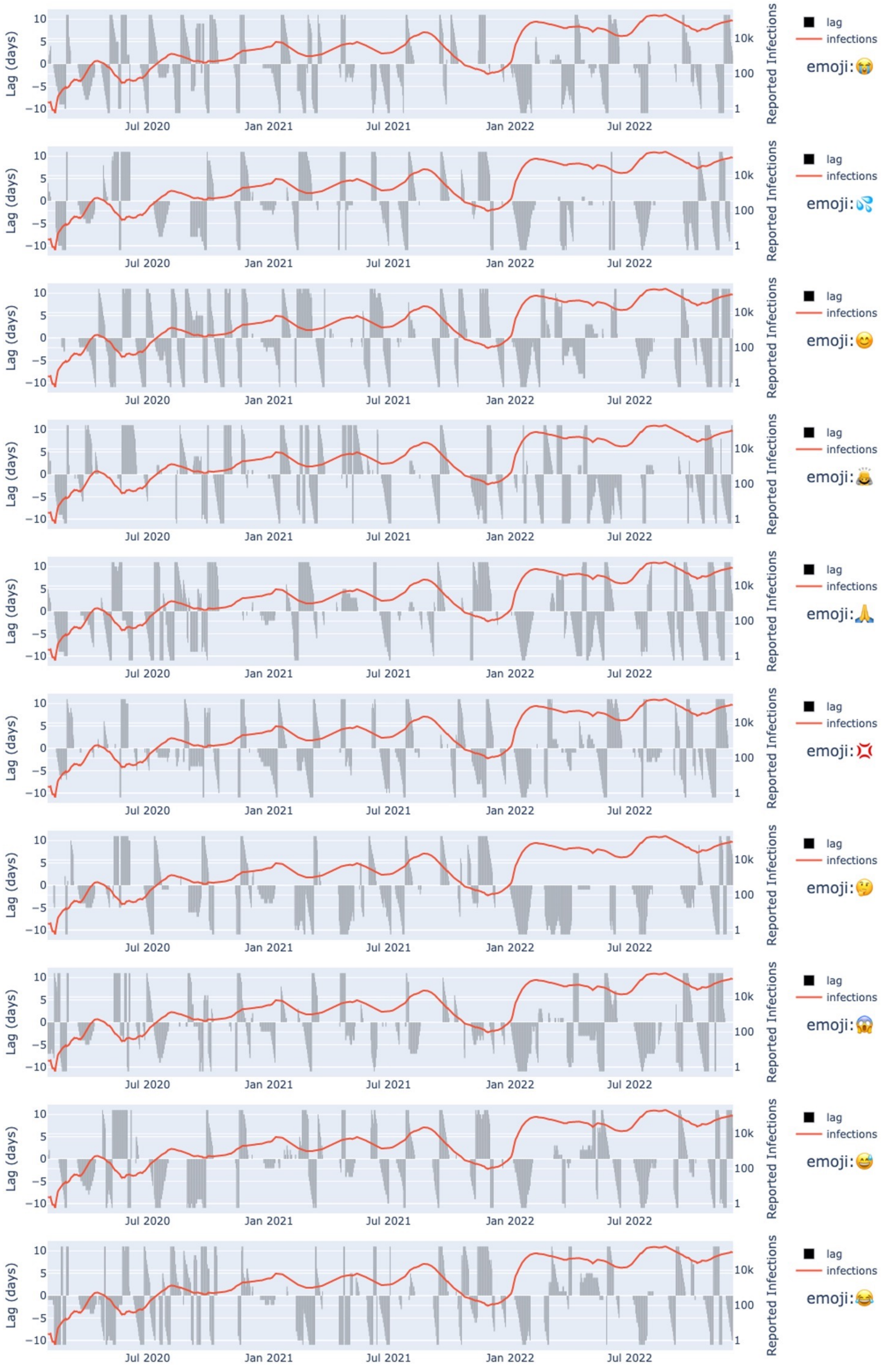

Figure S4. Cross-correlation between tweet count (using our defined keywords) and COVID-19 cases in Japan for top-10 used emoji.

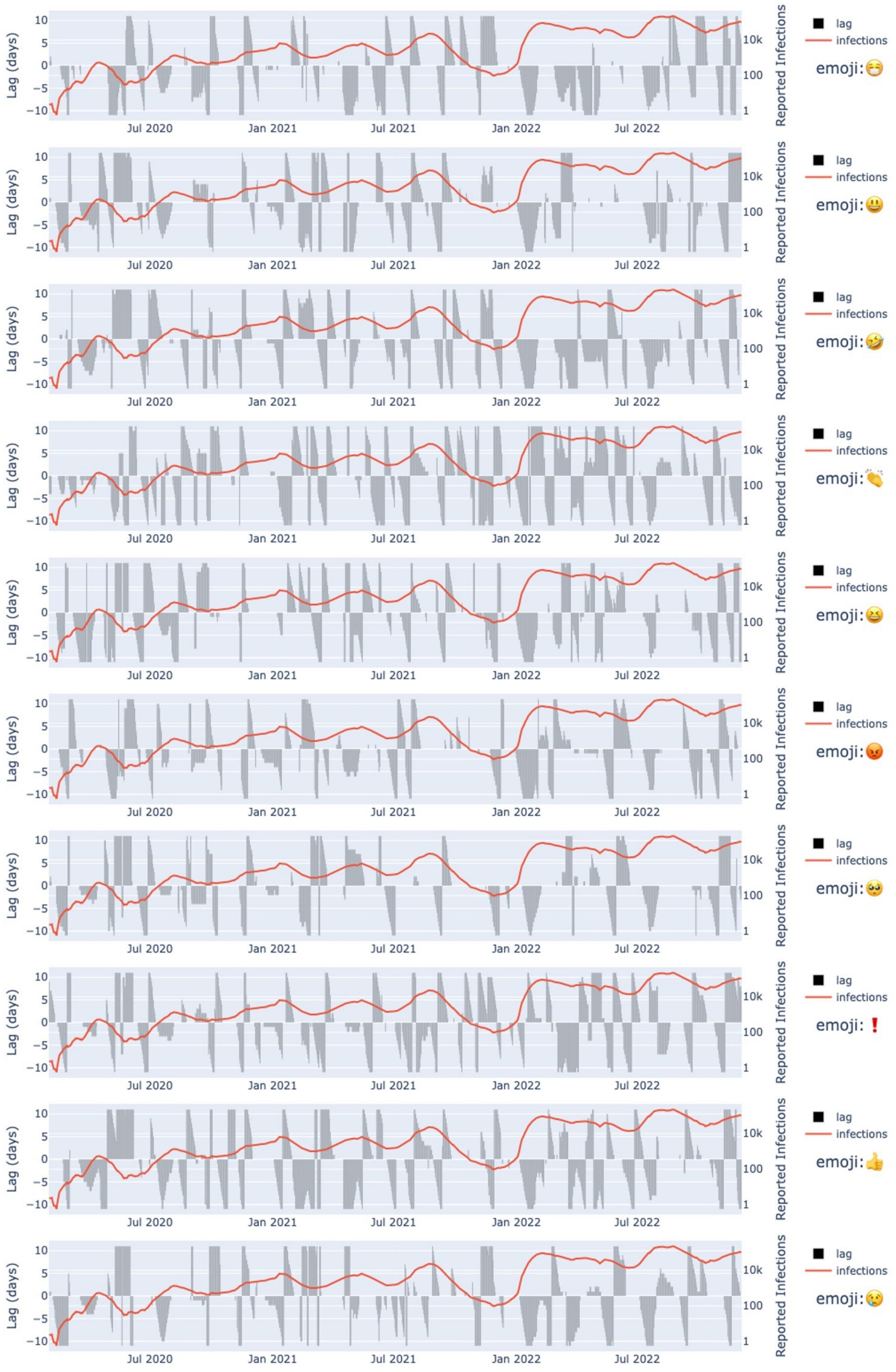

Figure S5. Cross-correlation between tweet count (using our defined keywords) and COVID-19 cases in Japan for 11th-20th used emoji.

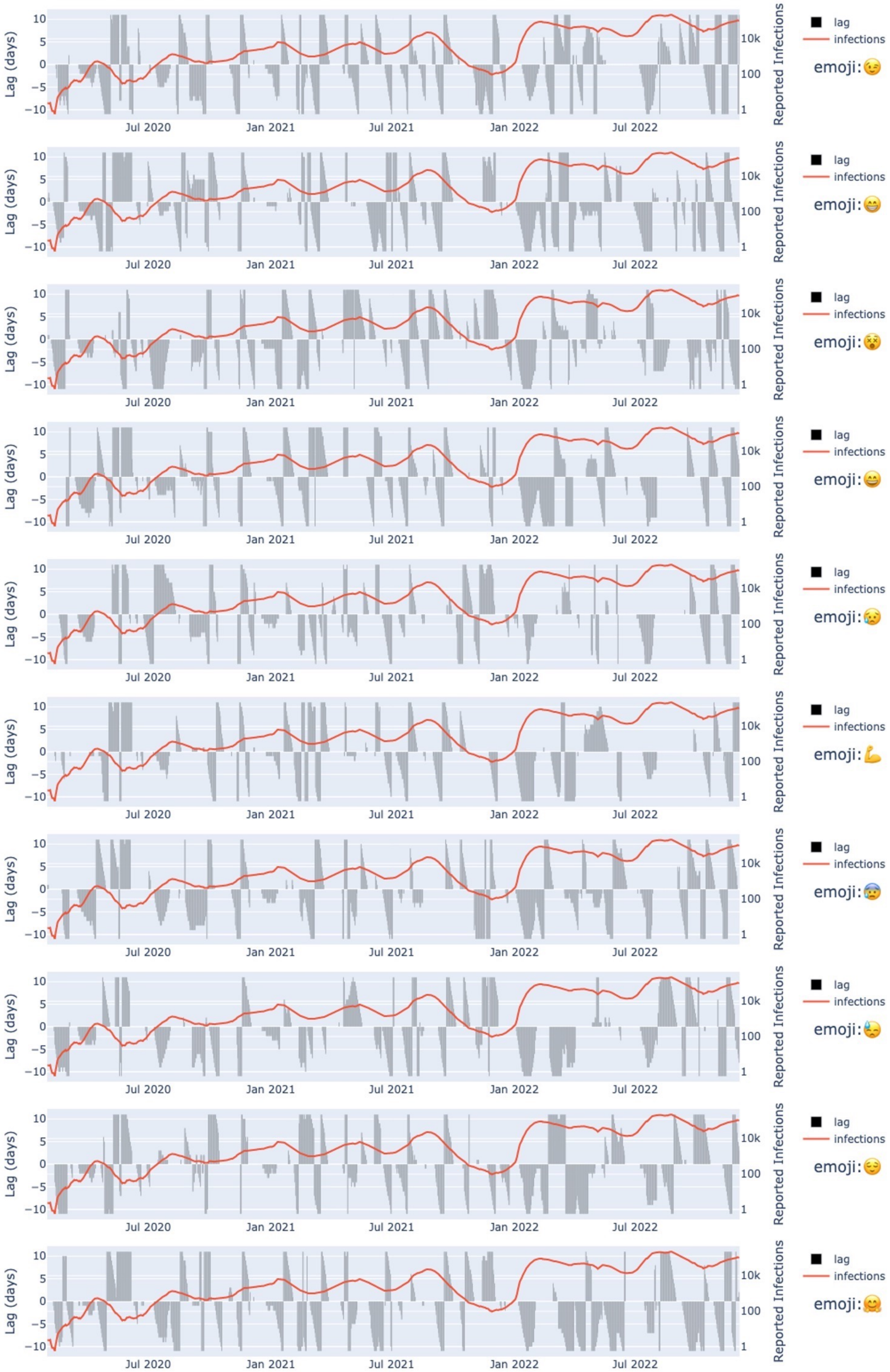

Figure S6. Cross-correlation between tweet count (using our defined keywords) and COVID-19 cases in Japan for 21st-30th used emoji.
